# Molecular Mechanisms of Ferroptosis and Its Roles in Hematologic Malignancies

**DOI:** 10.3389/fonc.2021.743006

**Published:** 2021-10-27

**Authors:** Yan Zhao, Zineng Huang, Hongling Peng

**Affiliations:** ^1^Department of Hematology, The Second Xiangya Hospital, Hunan Key Laboratory of Basic and Applied Hematology, Central South University, Changsha, China; ^2^Institute of Hematology, Central South University, Changsha, China; ^3^Hunan Key Laboratory of Tumor Models and Individualized Medicine, Changsha, China

**Keywords:** ferroptosis, regulated cell death, iron metabolism, reactive oxygen species, GSH, leukemia, lymphoma

## Abstract

Cell death is essential for the normal metabolism of human organisms. Ferroptosis is a unique regulated cell death (RCD) mode characterized by excess accumulation of iron-dependent lipid peroxide and reactive oxygen species (ROS) compared with other well-known programmed cell death modes. It has been currently recognized that ferroptosis plays a rather important role in the occurrence, development, and treatment of traumatic brain injury, stroke, acute kidney injury, liver damage, ischemia–reperfusion injury, tumor, etc. Of note, ferroptosis may be explained by the expression of various molecules and signaling components, among which iron, lipid, and amino acid metabolism are the key regulatory mechanisms of ferroptosis. Meanwhile, tumor cells of hematological malignancies, such as leukemia, lymphoma, and multiple myeloma (MM), are identified to be sensitive to ferroptosis. Targeting potential regulatory factors in the ferroptosis pathway may promote or inhibit the disease progression of these malignancies. In this review, a systematic summary was conducted on the key molecular mechanisms of ferroptosis and the current potential relationships of ferroptosis with leukemia, lymphoma, and MM. It is expected to provide novel potential therapeutic approaches and targets for hematological malignancies.

## 1 Introduction

Death is the common end of all life—from organisms to cells. The growth, development, balance, and stability of living organisms depend fundamentally on the organic balance of cell survival and cell death. Conventional cell death is an important physiological process in the growth and development of organisms or in the removal of excess or damaged cells to maintain the integrity of organisms. Therefore, cell death is crucial for normal development, homeostasis, and the prevention of hyperproliferative diseases such as tumors. As is known to all, cell death is mainly divided into two distinct categories of accidental cell death and regulated cell death (RCD). RCD is precisely regulated by genetic and signal transduction pathways primarily. It has been recognized to be the most important form of cell death involving apoptosis, autophagy, necroptosis, pyroptosis (1), and ferroptosis (**Table 1**).

**Table 1:** Features of different kinds of regulated cell death.

	Ferroptosis	Apoptosis	Autophagy	Necroptosis	Pyroptosis
Morphological feature	Small mitochondria with increased mitochondrial membrane densities and decreased volume, reduction and vanishing of mitochondria cristae	Plasma membrane blisters, cell and nuclear volume reduction, nuclear fragmentation	Formation of double-membraned autolysosome	Plasma membrane swollen, Organelle swelling, Moderate chromatin condensation	Karyopyknosis, cell edema and membrane rupture
Biochemical feature	Iron metabolism	DNA fragmentation	Increased lysosomal activity	Opening of PTPC	Dependent on caspase-1 and proinflammatory cytokine releases
	GSH deficiency	Caspase activation	P62 degradation	A drop in ATP levels	
	Lipid peroxidation				
Activation condition	Erastin	DNA damage	Nutritional deficiency	Severe oxidative stress	LPS
	RSL3	ROS overload death receptor activation	ER stress oxidative stress	Cytosolic Ca^2+^ overload	NLRP3 and other inflammatory bodies
	Sorafenib				
	I/R injury				
Key gene	GPX4, SCL7A11	Caspase-3, Bcl-2	ATG5, ATG7, LC3	CYPD, LEF1	Caspase-1, IL-1β
	P53, FSP1, ACSL4, VDAC2/3	Bax, Fas	Beclin 1	RIP1, RIP3, MLKL	IL-18, gasdermin D
	TFR1, FTH1, FTL	P53			
Relevant disease	Cerebral stroke, Cancer	Cancer, Viral infection	Cancer	Infection	Infection
	Ischemia–reperfusion injury	Autoimmune diseases	Parkinson disease	Toxins, trauma	Inflammation
	Acute kidney injury	Aplastic anemia	Cardiovascular disease		Diabetic nephropathy

CYPD, Cyclophilin D; LEF1, lymphoid enhancer-binding factor 1; ATG5, autophagy-related gene 5; ATG7, autophagy-related gene 7; MLKL, mixed lineage kinase domain like protein; LC3, microtubule-associated protein 1 light chain 3; RIP, receptor-interacting protein; Bcl-2, B-cell lymphoma 2; Bax, BCL2-Associated X; Fas, factor associated suicide; IL, Interleukin; LPS, Lipopolysaccharides; NLRP3, Nucleotide- binding oligomerization domain, leucine- rich repeat and pyrin domain- containing 3; VDAC, Voltage dependent anion channel; GSDMD, Gasdermin D.

Among them, ferroptosis is a newly discovered form of iron-dependent RCD induced by small molecules such as erastin (2) and Ras-selective lethal small molecule 3 [RSL3 (3)], as proposed by Dixon et al. (4) in 2012. It exhibits unique morphological, biochemical, and genetic features, which is different from those of the traditional form of cell death (4, 5). The characteristic morphological changes of ferroptosis are the shrunken mitochondria with ruptured external membrane, reduced or vanished cristae, condensed internal membrane, and intact cell nucleus (4, 6), while apoptosis and necroptosis generally have swollen mitochondria and broken nucleus (4). Furthermore, the biochemical mechanism of ferroptosis is mainly characterized by the production of lethal reactive oxygen species (ROS), lipid peroxidation, and iron accumulation intracellularly (7). It can further produce a large number of alkyl oxygen radicals, leading to fatal cell membrane damage and disorganization (8). In addition, genetically speaking, ferroptosis is a biological process regulated by multiple genes. It generally involves genetic changes in iron homeostasis and lipid peroxidation metabolism. Collectively, it highlights the importance of further investigation on corresponding underlying specific regulatory mechanisms (9).

Accumulated evidence has supported the critical role of ferroptosis in the development of tumors as the current research of relevant mechanisms going on. The existing treatment regimens, such as chemotherapy, immunotherapy, etc., may result in poor therapeutic efficiency for many hematological malignancies [e.g., leukemia, lymphoma, and multiple myeloma (MM)], suggesting an urgent need to explore new treatment modes. Herein, this review summarized the main molecular regulatory mechanisms of ferroptosis, with emphasis on its relationship with hematological malignancies. Our study is expected to provide a comprehensive understanding of ferroptosis and shed light on the development of novel therapeutic strategies for hematological malignancies.

## 2 Molecular Mechanisms of Cell Ferroptosis

The concept of ferroptosis was for the first time proposed in 2012 (4, 10, 11), referring to an iron-dependent mode of RCD caused by unrestricted lipid peroxidation and subsequent plasma membrane rupture. Ferroptosis can be normally induced by both endogenous and exogenous cellular pathways (12). The exogenous pathway is initiated by the inhibition of cell membrane transporters such as the cystine/glutamate reverse transporter (system Xc^-^) or by the induction of transferrin (TF) and ferroportin, which can contribute to the transportation of iron in and out of cells. The endogenous pathway can be further activated primarily by decreasing the expression or activity of intracellular antioxidant enzymes, such as glutathione peroxidase 4 (GPX4). At present, the core regulators of ferroptosis remain to be identified, since this process is known to be independent of the activity of caspases, mixed-lineage kinase domain-like protein (MLKL), or gasdermin D (13).

Specific small-molecule compounds act on the cellular anisotropic targets to cause the reduction of the antioxidant glutathione (GSH) or GPX4, resulting in intracellular ROS accumulation, lipid peroxidation, and induced cell ferroptosis under the synergistic effect of iron (5, 14). Free cystine can be transported intracellularly *via* the system Xc^-^, a substrate for GSH synthesis. Notably, GSH is a major redox molecule whose function is to protect against iron poisoning by donating an electron to GPX4 (15). More importantly, GPX4 is the only enzyme that reduces phospholipid peroxide (16). In addition, transferrin receptor 1 (TFR1), ferritin (FT), p53, ferroptosis suppressor protein 1 (FSP1), and lipoxygenase (LOX) are also involved in the occurrence of ferroptosis.

**Figure 1:**
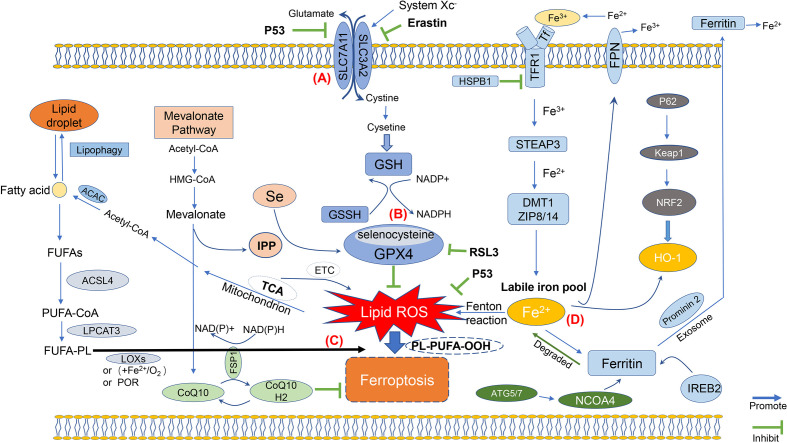
Molecular mechanisms of ferroptosis. The figure shows the molecular mechanisms of ferroptosis, which can be simply divided into four categories. **(A)** The first one is regulated by the system Xc; **(B)** Then, the second molecular regulatory mechanism is mainly the GSH/GPX4 pathway, which is currently one of the most important antioxidant systems; **(C)** Third, the molecular mechanisms of iron metabolism, such as IREB2 and Prominin 2, are related to ferritin metabolism, and both Ferroportin and TFR1 such as ACSL4 and LPCAT3, regulatory pathways have an impact on iron transport; **(D)** The fourth category is the lipid metabolism-related pathway, such as ACSL4, LPCAT3, which have an impact on lipid peroxidation and ferroptosis.

Collectively, ferroptosis is mainly caused by the intracellular imbalance between the production and degradation of lipid ROS (**Figure 1**). Oxidative cell death, namely, ferroptosis, can be caused when there is a reduced antioxidant capacity of cells or accumulation of lipid ROS. Importantly, the process of ferroptosis involves different signaling pathways, while the upstream pathway will, ultimately, lead to the occurrence of ferroptosis by directly or indirectly affecting the activity of glutathione peroxidases (GPXs), which may further reduce the antioxidant capacity of cells, and cause increased lipid peroxidation and lipid ROS.

### 2.1 Iron Metabolism

Iron is one of the important metal ions involved in various metabolisms in the human body and plays a vital role in promoting ferroptosis. Cellular iron metabolism consists of three main processes: iron uptake, storage, and output. Under physiological conditions, circulating free iron binds to TF, and iron metabolism *in vivo* maintains homeostasis. Fe^2+^ formed by intestinal absorption or erythrocyte degradation can be oxidized by ceruloplasmin to Fe^3+^. The generated Fe^3+^, which binds to TF on the cell membrane to form TF-Fe^3+^, is further transported into cells through TFR1 by constituting the TF-Fe^3+^/TFR1 complex to endocytose this complex (17). Heat shock protein B1 (HSPB1) can counter the increased expression of TFR1 and hence reduce the content of intracellular iron ions. Thus, an increase in HSPB1 expression can prevent ferroptosis (18, 19). In cells, Fe^3+^ is then reduced to Fe^2+^ again by iron reductases such as prostate six-transmembrane epithelial antigen of prostate 3 (STEAP3), which is then mediated by divalent metal transporter 1 (DMT1) for intracellular transmission (20). The remaining TF/TFR1 complex is eventually recycled to the cell surface for reuse. Fe^2+^ needs to be released into the labile iron pool (LIP), which is mediated by DMT1 or zinc–iron regulatory protein family 8/14 (ZIP8/14) (9). Under normal physiological conditions, the excess Fe^2+^ can be stored in two ways. It is either stored in FT to maintain iron levels or metabolized *in vivo* and even reoxidized by ceruloplasmin and recycled intracellularly (21). The proposed intracellular iron circulation tightly controls the homeostatic balance of iron use and recovery. Deletion of transferrin receptor protein 1 (*TFRC*), encoding TFR1, can inhibit ferroptosis induced by erastin (15), while the application of heme oxygenase-1 (HO-1) can block this effect by supplementing iron (22). More importantly, FT is the major protein for iron storage, which is composed of two ferritin heavy chain 1 (FTH1) and ferritin light chain (FTL) subunits (23). It has been reported that cancer cells with *ras* oncogene are more sensitive to ferroptosis, since *ras* can decrease the expression of *FTH1* and *FTL* to increase intracellular LIP. On the contrary, inhibiting the activity of iron-responsive element-binding protein 2 (IREB2), a regulator of ferroptosis, can enhance the expression of *FTL* and *FTH1* by preventing Erastin from inducing ferroptosis (24).

Recent studies have revealed that nuclear receptor coactivator 4 (NCOA4) is the selective autophagy flipping receptor of FT in ferroptosis (14, 25–28). The increase of intracellular iron caused by NCOA4-mediated degradation of FT is involved in ferroptosis. Cells treated with GPX4 inhibitors could secrete a large number of exosomes containing FT. During ferroptosis, the level of prominin2 is negatively correlated with the level of intracellular free iron, suggesting that exosomes can protect cells from ferroptosis by expulsing intracellular iron from cells (29, 30). Therefore, inhibition of prominin2 transcription can overcome ferroptosis resistance in cancer (31). Non-stored cytoplasmic iron is either transported to mitochondria for heme synthesis or incorporated in Fe-S clusters. Iron ions can act as cofactors of important enzymes in metabolic pathway, such as proline hydroxylase 2 (PHD2) and LOX. Intracellularly, free irons can regulate the posttranscriptional level of genes by affecting the binding of regulatory proteins iron regulatory protein (IRP)1 and IRP2 to iron response elements (IREs) within mRNAs. Moreover, IRPs can adjust the expression of proteins that may affect the movement of intracellular iron ions into and out of cells [TFR1, DMT1, ferroportin (FPN) (32)], storage (FTH), and utilization according to iron metabolism in cells (33).

The excess Fe^2+^ can generate lipid ROS by the Fenton reaction (6). To be precise, the Fenton reaction is an *in vivo* metabolic response between Fe^2+^ and hydrogen peroxide (H_2_O_2_) to generate chemicals capable of oxidizing various organic substrates. Under the action of Fe^2+^, the Fenton reaction converts H_2_O_2_ to hydroxyl radical to promote further free radical chain reaction. However, it has been shown that radical-trapping antioxidants (RTAs) can provide electrons to neutralize free radicals (34), thereby inhibiting lipid peroxidation of cell membranes. The classical Fenton reaction between Fe^3+^ and Fe^2+^ can produce hydroxyl radicals that can damage membrane, DNA, lipids, and proteins, which will eventually cause ferroptosis (35). Therefore, the regulation of iron metabolism may also be a potential direction for mediating ferroptosis.

### 2.2 Lipid Metabolism and Reactive Oxygen Species Accumulation

It is well known that all ferroptosis-promoting pathways in abnormal cells can ultimately contribute to the production of lipid ROS to induce cell membrane destruction. Iron-dependent lipid ROS, produced by normal physiological processes *in vivo*, can significantly affect cell signal transduction and tissue homeostasis. However, excessive ROS-induced lipid peroxidation can adversely modify cell components, such as lipid, protein, and DNA damage (36). Biological cell membranes or organelle membranes are particularly vulnerable to ROS damage, since they are rich in high polyunsaturated fatty acids (PUFAs), which are known as lipid peroxidation (37, 38). With double bonds near the diallyl methylene, PUFAs are more readily oxidized than saturated fatty acids (SFAs) and monounsaturated fatty acids (MUFAs). Lipid peroxidation can destroy the lipid bilayer and affect membrane function (39). Free PUFAs are organic substances that synthesize lipid signal transduction mediators, while their formation of membrane phospholipids depends on their combination by some compounds and their oxidation is the premise to transmit ferroptosis signals (9). A recent liposome-logical study showed that when PUFAs contain arachidonic acid (AA) and adrenaline (ADA), there would be a higher possibility of the oxidation of proteoliposomes (PLs) on the cell membrane, especially phosphatidylethanolamine (PEs). Excessive oxidation can eventually cause ferroptosis (40). Moreover, PEs are a type of key phospholipid molecules that induce ferroptosis intracellularly, since they contain AA or their derivative epinephrine (25, 41). The enzymes, i.e., acyl-CoA synthetase long-chain family member 4 (ACSL4) and lysophosphatidylcholine acyltransferase 3 (LPCAT3) (39, 42), are required for the biosynthesis and remodeling of PEs on the cell membrane during the initiation of lipid peroxidation, which can activate PUFAs and affect the transmembrane properties of PUFAs. Therefore, the production of membrane phospholipid biosynthetic enzymes regulating PUFAs may influence the development of ferroptosis. Accordingly, decreasing the expression levels of ACSL4 and LPCAT3 can reduce the accumulation of lipid peroxide substrates intracellularly, thereby inhibiting ferroptosis. Two enzymes, ACSL4 and LPCAT3, are promising in preventing ferroptosis or other peroxidation-related diseases. By contrast, ACSL3 can convert MUFAs into their acyl-CoA esters for incorporation into membrane phospholipids, thus protecting cancer cells against ferroptosis. LOX is an iron-containing enzyme used for the oxidation of membrane PLs. Identically, it is also a non-heme dioxygenase that can catalyze the peroxidation of free and esterified PUFAs, resulting in the occurrence of ferroptosis (35, 43, 44). Elimination of LOX gene can inhibit ferroptosis damage induced by erastin (7). Ultimately, PUFA-PE is further oxidized by LOX catalysis and causes ferroptosis in a wide range of cell types (41).

### 2.3 Glutathione Peroxidase 4

GPX4 is an antioxidant enzyme belonging to the GPX family and one of the 25 proteins containing selenocysteine (Sec) in the human genome 17 (37). Using GSH as a reaction substrate, GPX4 can stimulate its conversion into oxidized glutathione (GSSG) and reduce lipid hydrogen peroxide (LOOHs) to the corresponding non-toxic lipid alcohols (L-OH). The consumption of two GSH molecules in this reaction ultimately prevents the synthesis of lipid peroxides (45). However, the production of LOOHs may increase with the decrease of GPX4 expression, leading to the damage to the membrane by lipid peroxidation (46, 47), which is a hallmark of ferroptosis. Thus, GPX4 may act as a central repressor of ferroptosis in cancer cells. Covalent binding of GPX4 and the blockage of its expression are available to directly inhibit GPX4. Previous studies have reported that GPX4 is a protein target of RSL3. The compound RSL3 (3) can specifically inhibit GPX4 activity and lead to intracellular ROS accumulation through covalently binding to the Sec at the active site of GPX4, thus inducing ferroptosis (46, 48).

Friedmann Angeli et al. (6) demonstrated that intracellular ferroptosis could be induced through knocking out GPX4 gene. In addition, the compounds DPI7 and DPI10 can also directly inhibit the action of GPX4 to promote ferroptosis. Selenium is a necessary micronutrient with multiple antioxidant capabilities, which is beneficial to human health. It is mainly incorporated into selenoprotein in the form of Sec and is an important component of GPX4 (49). Therefore, selenium can affect cell sensitivity to ferroptosis to some extent (50). Furthermore, selenium can enhance GPX4 and other genes in this transcription program through the synergistic activation of transcription factors transcription factor activating protein 2 gamma (TFAP2C) and specificity protein 1 (SP1), effectively inhibiting GPX4-dependent ferroptosis, while selenium deficiency will inactivate GPX4, leading to increased sensitivity of cells to oxidative damage (51, 52). Moreover, FINO_2_ and FIN56 can induce ferroptosis by indirectly inhibiting GPX4 levels and activity without affecting GSH levels (53, 54). The mevalonate (MVA) metabolic pathway is responsible for the synthesis of the precursor of synthetic steroids and other biomolecules with acetyl-CoA as raw materials, which can produce isopentenyl pyrophosphate (IPP) and coenzyme Q (CoQ)10 (49, 54). CoQ10 is an endogenous antioxidant of cells. It has an antioxidant effect on the cell membrane and inhibits ferroptosis by blocking the lipid peroxidation process. Ferroptosis-inducing agents (FINs) of ferroptosis on GPX4 can consume CoQ10 through the MVA pathway and decrease the expression of GPX4 protein, thus enhancing the accumulation of lipid peroxidation and causing ferroptosis.

### 2.4 System Xc- and Glutathione Production

Amino acid metabolism is closely related to the regulation of ferroptosis (55). System Xc^-^, an important antioxidant system, is an amino acid anti-transporter located in the cell membrane and comprises a heterodimer composed of two subunits, i.e., solute carrier family 3 member 2 (SLC3A2) and solute carrier family 7 member 11 (SLC7A11) (56, 57). Cystine and glutamate are transported in and out of cells mediated by system Xc^-^ and exchanged in a 1:1 ratio for the synthesis of GSH (48, 58, 59). Cystine is reduced to cysteine to participate in GSH synthesis by system Xc^-^ (60). The transport process of this system does not require ATP, and it is mediated by the difference in the concentration of amino acids presenting on both sides of the cell membrane (61). GSH is a tripeptide comprising glutamate, cysteine, and glycine. Among them, cysteine is presented in relatively small amounts in cells and is therefore considered to be a dominant condition governing GSH synthesis. The natural synthesis of GSH is critical for GPX4 to exert its biological activity. In a GPX-catalyzed chemical reaction, the GSH synthesis can be inhibited by decreasing ROS levels, leading to oxidative damage and cell death (56). It has been demonstrated that this classical cellular oxidative stress pathway is associated with ferroptosis. GPX4 converts two molecules of GSH to GSSG (62) and simultaneously reduces LOOH to phospholipids-H (L-OH) to reduce the membrane accumulation of toxic substances (62, 63). By inhibiting the uptake of cystine, it can suppress the activity of system Xc^-^ and prevent the synthesis of GSH, which may in turn weaken GPX activity, a decrease in cellular antioxidant capacity, the accumulation of lipid peroxides, and ultimately oxidative damage and ferroptosis in cells (42). In addition, the oncogene P53 can also inhibit the transport of cystine in system Xc^-^ by downregulating the expression of SLC7A11, which consequently elevates the sensitivity of cells to ferroptosis and thus affects the activity of GPX4, leading to a decrease in the antioxidant capacity of cell membranes and cell death by destruction (64, 65). Overall, the intracellular transport of cysteine through the system Xc^-^ is important for affecting cysteine and GSH levels, and hence inhibiting the onset of ferroptosis. In case of abnormality, the level of GSH will be reduced that may produce a negative effect on GPX4 to exert its inhibitory roles of cell peroxidation and death.

### 2.5 p53

The TP53 gene is an essential tumor suppressor gene for humans. It is generally believed that p53-mediated cell cycle arrest, apoptosis, and senescence are the major causes explaining tumor suppression (66). However, it remains unclear with regard to the mechanism of TP53 gene in ferroptosis. It has been reported recently that p53-mutants lacking acetylated modifications can promote ferroptosis. Jiang et al. (65) reported that SLC7A11 was overexpressed in multiple human cancers, and p53 could reduce cystine uptake by inhibiting SLC7A11 transcription, decrease intracellular GSH, and increase intracellular ROS accumulation, thereby increasing the susceptibility of cells to ferroptosis. Analysis based on a mutant mouse model showed that the changes in atypical p53 activity could benefit the understanding of the development and mortality of embryos associated with murine double mimute 2 (MDM2) deficiency. The regulation of ROS levels by p53 is an interesting process. At low or basal intracellular ROS levels, p53 can prevent cells from accumulating lethal levels of ROS; while in the case of an abnormally high ROS levels, however, p53 may promote cell clearance through ferroptosis. Therefore, p53 may have a regulatory role in ferroptosis by affecting intracellular ROS levels. This finding may suggest a novel tumor suppression model based on p53 to regulate cystine metabolism, ROS response, and ferroptosis. In addition, ferroptosis can also be inhibited through the P53–P21 axis under certain circumstances. Meanwhile, as mentioned before, ferroptosis is mainly mediated by GPX4. Surprisingly, p53 activation was found to modulate ferroptosis (64, 65, 67) but had no significant effect on GPX4 function, while Chu et al. (68) found that arachidonate 12-lipoxygenase (ALOX12) (68, 69) inactivation attenuated p53-mediated ferroptosis induced by ROS substances and promoted the rapid growth of p53-dependent tumors in xenograft tumor models, suggesting that ALOX12 gene may be critical for p53-mediated ferroptosis.

### 2.6 Other Mechanisms

Besides the above pathways, there are several other metabolic factors that can regulate cell sensitivity to ferroptosis. Two recent studies have reported the role of FSP1 in the development and progression of ferroptosis, demonstrating a novel and effective method to regulate ferroptosis with the reintroduction of apoptosis-inducing factor (AIF) (70, 71). Nicotinamide adenine dinucleotide phosphate (NADPH) is a glutathione reductase that reduces GSH to regulate ferroptosis (7). Therefore, NADPH can be a potential biomarker to determine whether ferroptosis inducers are sensitive to cancer cells (72). Furthermore, NADPH oxidase (NOX)-mediated bio-oxidation is a significant pathway for lipid free radical production. Overexpression of NOX, an enzyme complex, can lead to depletion of NADPH and elevated levels of oxidative free radicals, which significantly increases the sensitivity of cells to ferroptosis. In addition, NOX can act in three approaches. Firstly, p53 impedes ferroptosis in colorectal cancer (CRC) cells by binding to DPP4, which is associated with NOX1 (73). Secondly, AA significantly increased the phosphorylation level of NOX (74) mediated by protein kinase C. It may further result in increased NOX phosphorylation, which increases the amount of oxidative free radicals and the risk of ferroptosis. Finally, the Hippo (75–78) pathway is also responsible for the occurrence of ferroptosis. Luo et al. (79) observed that miR-137 can exert antitumor effects by modulating the 3°C untranslated region (UTR) of SLC1A5 (a major glutamine transporter) mRNA to regulate ferroptosis.

It is well known that GPX4 and FSP1 constitute two major defenses against ferroptosis. The main mechanisms are described as follows: 1) GPX4 reduces toxicity caused by lipid hydroperoxides by reduced GSH; and 2) FSP1 is a GSH-independent ferroptosis inhibitor. As an oxidoreductase, it reduces CoQ on the cell membrane to panthenol (CoQH2), which traps free radicals and inhibits lipid peroxides by acting as a lipophilic antioxidant. However, a recent study reveals a third mechanism by which ferroptosis is inhibited (80). It reported that treatment of cancer cells with GPX4 inhibitors resulted in rapid depletion of N-carboxyl-L-aspartic acid, a pyrimidine biosynthesis intermediate, accompanied by the production of uridine. Besides, supplementation with the substrate and product of dihydroorotate dehydrogenase (DHODH) attenuated or enhanced the inhibition of ferroptosis induced by GPX4, respectively, especially in cancer cells with low GPX4 expression (GPX4^low^). DHODH inactivation could induce extensive mitochondrial lipid peroxidation and ferroptosis in GPX4^low^-expressing cancer cells, while in GPX4^high^-expressing cancer cells, these effects could be induced simultaneously by synergistic action with ferroptosis inducers. Collectively, the aforementioned findings confirm DHODH-mediated ferroptosis defense mechanism in mitochondria, suggesting a therapeutic strategy for the targeted treatment of ferroptosis in tumors.

## 3 Application of Ferroptosis in Hematologic Malignancies

Ferroptosis has been proven to be extensively involved in multiple system diseases, such as nervous system diseases (81), heart diseases, liver diseases (82), gastrointestinal diseases (73, 83), lung diseases (84), kidney diseases (6, 85, 86), and pancreatic diseases (87). As for the blood system, ferroptosis has been disclosed to play an important role in many hematological neoplasms such as leukemia. Especially in the research field of cancers, hematological malignancies may share a similar mechanism with solid tumors in ferroptosis.

**Figure 2:**
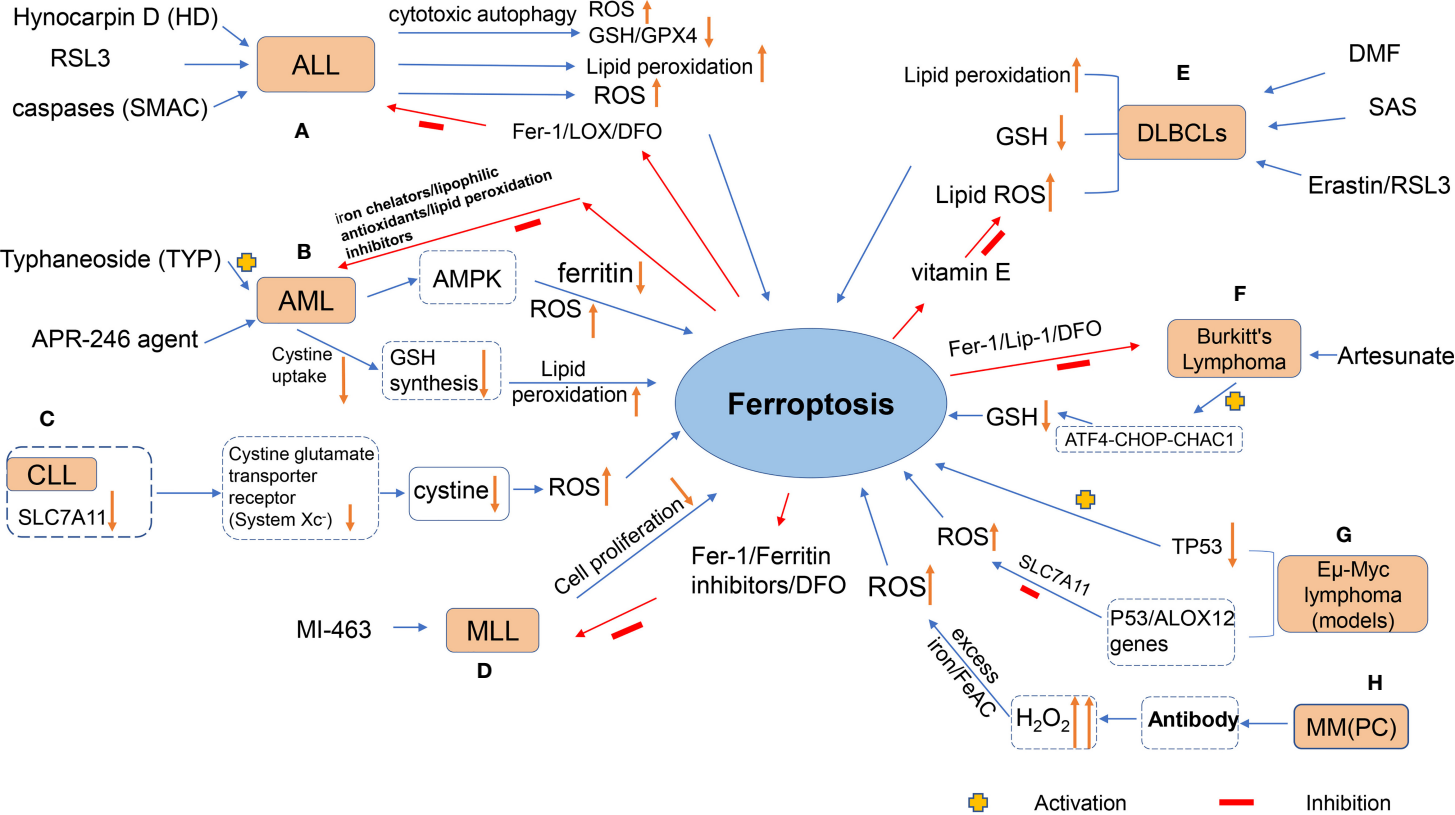
The figure shows the agents or mechanisms that regulate ferroptosis in animal models or patients with hematological malignancies. **(A)** Treatment of ALL cells with HD, RSL3 and SMAC resulted in ferroptosis with increased lipid peroxidation levels, which was inhibited by antioxidants and DFO; **(B)** TYP treatment of AML cells could lead to ferroptosis in AML cells through activation of AMPK signaling, accompanied by ferritin degradation and ROS accumulation; APR-246 action on AML cells decreased cystine uptake, resulting in decreased GSH synthesis and lipid peroxidation in the cell membrane (this effect can be inhibited by iron chelators, lipophilic antioxidants and lipid peroxidation inhibitors); **(C)** Expression of SLC7A11 would definitely be down-regulated in CLL cells, and system Xc- transporting cystine capacity could be reduced, leading to increased intracellular ROS and promoting cellular ferroptosis; **(D)** MI-463 action on MLL led to the inhibition of tumor cell proliferation by ferroptosis of its cells, which could be reversed by the use of ferroptosis inhibitors. **(E)** Treatment of DLBCLs with RSL3, Erastin, and DMF resulted in ferroptosis of cells accompanied by increased lipid peroxidation levels, increased ROS, and decreased GSH levels, which was inhibited by antioxidants, DFO, and vitamin E; **(F)** Artesunate induced ferroptosis in Burkitt‘s lymphoma(BL) cells by activating the ATF4-CHOP-CHAC1 pathway and degrading GSH; **(G)** p53 inhibited system Xc- and promoted ferroptosis in *Eμ-Myc* lymphoma cells, while deletion of TP53 gene accelerated the formation of *Eμ-Myc* lymphoma model; **(H)** MM could generate lipid ROS through high levels of H_2_O_2_, a byproduct of antibody production, which reacted with excess iron in the Fenton reaction, thereby destroying the cell membrane and causing cell death.

Hematological malignancies, or fluid tumors vividly, are a category of cancers that originate from cells of the hematopoietic system (such as bone marrow) or those of the immune system. A single germline or somatic mutation in lymphatic hematopoietic stem cells may be prone to clonal expansion, depending on the acquired new mutation (88), which is commonly malignant and may result in an undesirable outcome, i.e., tumors. For instance, lymphomas arise from lymphocytes at different stages of development, and their subtype characteristics, B-cell and T-cell tumors, reflect the cells of origin (89). Leukemia, on the other hand, is a heterogeneous group of diseases characterized by clonal expansion, abnormal proliferation of undifferentiated myeloid or lymphoid progenitors, and variable responses to treatment (90). Moreover, MM is a malignant plasma cell (PC) disorder originating from PCs formed by B-lymphocyte development in the bone marrow (91). Therefore, its pathology is characterized by abnormal proliferation of bone marrow PC, with overproduction of monoclonal immunoglobulin or light chain (M protein). There is growing evidence that ferroptosis may be a driving factor in these hematologic tumors, which are caused by hematologic dysfunction and cell death. **Figure 2** shows the agents or mechanisms that regulate ferroptosis in animal models or patients with these hematological malignancies.

Common hematological malignancies mainly include various types of leukemia, malignant lymphoma, and MM as stated above. Chemotherapy, immunotherapy, and hematopoietic stem cell transplantation (HSCT) are common choices for the treatment of these malignancies (92–98). Stem cell transplantation therapy still has certain limitations in its clinical application, despite great improvement achieved in recent decades. Furthermore, the most substantial advances have been made in the use of chemotherapy for the treatment of hematologic malignancies in the last decades. It achieves high remission rates by using chemotherapy regimens; the cure rates, however, are not high, which seriously reduce the patients’ survival and life quality (99). Therefore, there is still a need to explore additional treatment options that will benefit patients more, such as targeted therapy. Excitingly, it has been discovered through mediating ferroptosis, there may be a possibility to kill tumor cells in the blood system (100) and prevent tumor progression. Ferroptosis, a newly reported cell death, has attracted great attention in the field of tumor research and treatment (101, 102). At present, the research on ferroptosis in hematological tumors emphasizes on regulating ferroptosis pathway-specific molecules to affect the sensitivity of cells to ferroptosis, so as to regulate the progression of ferroptosis of tumor cells and reach the therapeutic aim. We will elaborate on the relationship between ferroptosis and these hematological malignancies in detail below.

### 3.1 Leukemia

#### 3.1.1 Acute leukemia

##### 3.1.1.1 Acute Lymphoblastic Leukemia

Acute lymphoblastic leukemia (ALL) is a malignant neoplastic disease with abnormal proliferation of lymphocyte B or T cells in the bone marrow. Abnormal proliferation of primitive cells can be aggregated in the bone marrow and inhibit the normal hematopoietic function. Moreover, it can invade the tissues outside the bone marrow, such as meninges, lymph nodes, gonads, and liver. Hynocarpin D (HD) is a bioactive flavonoid lignin compound with good antitumor activity but insufficient understanding of its mechanism. Lou et al. (103) found that in T-cell acute lymphoblastic leukemia (T-ALL) cell lines Jurkat and Molt-4, HD could inhibit the proliferation of T-ALL by inducing cell cycle arrest and subsequent apoptosis. In addition, HD could also increase LC3-II levels and the formation of autophagy lysosomal vacuoles, both of which are markers of autophagy. Autophagy was inhibited by ATG5/7 or pretreated 3-MA, accompanied by partial preservation of HD-induced apoptosis, suggesting that autophagy enhanced the efficacy of HD. Moreover, this study also found that such cytotoxic autophagy could induce ferroptosis and exert an antitumor role, as evidenced by the accumulation of lipid ROS and reduction of GSH and GPX4, while the inhibition of autophagy could impede ferroptosis. In another study by Probst et al. (104) using ALL cell lines, treatment with RSL3 resulted in the death of the experimental cell lines, accompanied by an increase in the level of lipid peroxidation. The addition of lipid peroxidation inhibitor Fer-1 or LOX could inhibit cell death. Moreover, iron-chelating agent deferoxamine (DFO) could reverse RSL3-induced ferroptosis, suggesting that ALL cells are sensitive to RSL3-induced ferroptosis. Besides, Dächert et al. (105) reported that the cell death simulated by the second mitochondrial activator of caspases (SMAC) was regulated by the redox signal. In addition, RSL3, a GPX4 inhibitor, or erastin, a cystine/glutamate antiporter inhibitor, could work with SMAC to mimic BV6 to induce ROS-dependent cellular ferroptosis in ALL cells.

The above studies have shown that ALL is highly sensitive to ferroptosis (103–107), accompanied by excessive accumulation of ROS and increased lipid peroxidation level, which is consistent with the currently known classical pathway of ferroptosis. Overall, these studies offer novel insight into the molecular regulatory mechanisms of ferroptosis and may contribute to developing new therapeutic strategies to reactivate programmed cell death in ALL.

##### 3.1.1.2 Acute Myeloid Leukemia

Acute myeloid leukemia (AML) includes all acute leukemia of non-lymphoid origin. It belongs to a malignant clonal proliferative disease of myeloid primitive cells in the hematopoietic system. In general, it is also a highly heterogeneous disease that can be transformed by the malignant transformation of hematopoietic progenitor cells at different stages of normal myeloid cell differentiation and development. The incidence of AML increases with age, and >90% of deaths occur after the age of 65 (108). To our knowledge, the molecular mechanisms underlying disease progression and clinical prognosis of AML are generally attributed to genetic, epigenetic, and proteomic alterations (109). At present, the research on ferroptosis in AML has been a hot topic, providing novel and valuable insights for the application of ferroptosis-promoting compounds in the treatment of hematologic tumors including AML (**Table 2**). Our subsequent content will give an introduction about the specific application of ferroptosis in AML by examples from existing studies.

**Table 2:** Research progress on ferroptosis in acute myeloid leukemia.

Researcher	Research object	Related Pathway	Target	Conclusions/Results
Sarah Weber (110)	Patients/mice	Iron Metabolism pathway	Iron	Iron overexpression in patients with MDS and AML
Eric Grignano (111)	Leukemic cells *in vivo* experiments/Patients	Iron Metabolism pathway	Iron	Iron overload in AML
Rudy Birsen (112)	AML cells both *in vivo* and *ex vivo*	GSH synthesis pathway	System Xc^-^ GPX4	SLC7A11 or GPX4 genes were inactivated in MDS and AML cells after exposure to APR-246
Jie Wei (113)	AML patient samples and normal samples	Lipid peroxidation	Glutathione peroxidases (GPXs)	High expression of GPX-1, -3, -4, and -7 in AML patients
Yan Du (114)	AML xenograft mouse model/*in vitro* studies	Lipid synthesis pathway	ROS	Overexpression of lipid ROS in AML cells due to ATPR
Hai-Yan Zhu (115)	AML cells	Lipid synthesis pathway	ROS	The accumulation of intracellular and mitochondrial ROS and the activation of AMPK in AML due to TYP
Rushdia Zareen Yusuf (116)	Leukemic cells in multiple mouse/human myeloid leukemias	Lipid peroxidation	Glutathione peroxidase-4 (GPX4)	Glutathione peroxidase-4 (GPX4) inhibitors inhibited ALDH3A2 in AML
Fanghua Ye (117)	HL-60/NRAS ^Q61L^ cells	The RAS-JNK/p38 pathway	TFR1	Through the Ras-JNK/p38 pathway, HMGB1 was downregulated and reduced the level of TFR1 in HL-60 cells
Jing Du (118)	AML cell lines	AMPK/mTOR/p70S6k signaling pathway	Ferritin/Iron/ROS	Increased unstable iron and ROS in AML due to DHA	
Jessica Sagasser (119, 120)	Leukemia cell lines such as HL-60 cells	Lipid synthesis pathway	ROS	Chloride [N,N'-disalicidene-1,2-phenylenediamine] iron(III) complexes produced, induced lipid ROS and induce ferroptosis in HL-60 cells	
Yan Yu (121)	HL-60 cells	Activation of JNK and p38 signaling pathway	HMGB1 release	Erastin increased the sensitivity of AML cells to chemotherapeutic agents.	
Li-Hua Dong (122)	AML cell lines(K-562 and HL-60)/ the nude mice/AML patients	The putative binding sites among circKDM4C, hsa-let-7b-5p, and P53	P53	CircKDM4C upregulated P53 by sponging hsa-let-7b-5p to induce ferroptosis in AML	
Zhe Chen (123)	AML cells	Iron metabolism pathway	Iron	Increased iron ameliorated AML by inducing the death of iron-dependent cancer cells	

AML has been currently found to be sensitive to compounds that promote ferroptosis. Typhaneoside (TYP) is the main flavonoid in the extract of Pollen Typhae, suggesting important biological and pharmacological effects. For example, with the use of TYP for the treatment of AML cells, there were significant increases in intracellular and mitochondrial ROS levels; simultaneously, TYP induced ferroptosis in AML cells in an iron-dependent manner, accompanied by mitochondrial dysfunction. In addition, TYP significantly triggered autophagy in AML cells by promoting the activation of AMP-activated protein kinase (AMPK) signals, resulting in the degradation of FT, ROS accumulation, and ultimately ferroptosis of cells (115). Taken together, this study provides conclusive evidence that TYP could be a potential therapeutic agent to prevent the progression of AML by inducing cellular ROS production and ferroptosis.

Notably, p53-mutated proteins were found in patients with myelodysplastic syndrome and AML in earlier studies. As a promising novel therapeutic agent, APR-246 can inhibit the proliferation of cancer cells by promoting the binding of p53 mutants to DNA targets sites and reactivating their transcriptional activity. Studies in solid cancers have shown that APR-246 can also induce p53-independent cell death. In 2021, Birsen et al. (112) observed that early AML cell death after exposure to APR-246 was inhibited by iron-chelating agents, lipophilic antioxidants, and lipid peroxidation inhibitors, leading to aberrant accumulation of lipid peroxides, confirming ferroptosis. Therefore, cells exposed to APR-246 can maintain GSH biosynthesis by increasing cystine uptake and ultimately prevent cells from producing lipid peroxides. These results firmly confirm that APR-246 induces early cell death in AML through ferroptosis and that APR-246 can synergistically promote cell death with ferroptosis inducers, whether through drug compounds or gene inactivation of SLC7A11 or GPX4, both *in vivo* and *in vitro*.

To recap, the current research on AML generally includes the development of new therapeutic reagents that produce interference to a certain link in the molecular mechanism of ferroptosis, so as to kill tumor cells and alleviate disease progression. However and importantly, due to a limited research in this field, further studies should be conducted to reveal the specific mechanisms underlying the role of ferroptosis in AML.

##### 3.1.1.3 Mixed-Lineage Leukemia-Rearranged Leukemias

It has been recognized that genetic or environmental changes are the common causes for the occurrence of different types of leukemia. Anyway, there is still a need for further elaboration of the exact molecular mechanisms of these heterogeneous diseases (124). Of note, mixed-lineage leukemia (MLL) gene is located on chromosome 11q23, and rearrangement of this gene is a common change in hematopoietic malignancies (125). MLL gene rearrangement-positive acute leukemia (AL) can occur from birth to adulthood, presenting as ALL or AML. These leukemia patients are found with unique clinical and biological characteristics, including high white blood cell count, insensitivity to conventional chemotherapy, low complete response rate (CR), short survival rate, and worse prognosis in patients younger than 1 year of age (126). Therefore, it has been classified as 11q23/MLL leukemia, a special subtype of leukemia, as defined by the World Health Organization.

In 2020, Kato et al. (127) reported the potential of MLL inhibitors in the treatment of MLL-rearranged leukemias. The use of menin-MLL inhibitors, such as MI-463, could unexpectedly induce ferroptosis in leukemia cells. Ferrostatin 1 (an inhibitor of ferroptosis) almost eliminated the MI-463-induced reduction in the number of living cells, while the effect of Z-VAD-FMK (an inhibitor of apoptosis) on cell death could be negligible. Both FT inhibitors and DFO could eliminate the synergistic induction of cell death. Hence, menin-MLL inhibitors (e.g., MI-463) may be an effective method for the treatment of MLL by inducing ferroptosis.

#### 3.1.2 Chronic Leukemia

##### 3.1.2.1 Chronic Lymphocytic Leukemia

Chronic lymphocytic leukemia (CLL) is a heterogeneous disease in terms of its genetic characteristics and response to treatments. It is characterized by an accumulation of monoclonal B cells (CD20^+^, CD5^+^, and CD23^+^) in the peripheral blood, bone marrow, and secondary lymphoid organs, resulting in the failure of the immune system. Additionally, despite a slow disease progression, CLL is especially difficult to cure. Few studies report ferroptosis in CLL so far, and the example is as follows.

Human CLL cells cannot convert methionine to cystine, and therefore extracellular cystine uptake is essential for their growth and progression. Compared with the high expression of SLC7A11 in other systemic solid tumors, its expression is downregulated in CLL, accompanied by a reduced ability of system Xc^-^ to transport cystine, which can promote the increase of intracellular ROS, resulting in membrane lipid peroxidation and hence cell death. It may suggest an intimate association of CLL with ferroptosis (128).

### 3.2 Lymphoma

#### 3.2.1 Diffuse Large B-Cell Lymphoma

DLBCL is the most common hematologic malignancy. It is characterized by diffuse proliferation of large B cells, and its tumor nucleus is at least twice that of normal lymphocytes. The common clinical symptoms of DLBCL are painless and progressive lymphadenopathy as well as extranodal mass. Despite the emergence of new targeted agents currently, molecular heterogeneity of DLBCL remains to be a major therapeutic challenge. **Table 3** summarizes the existing studies on ferroptosis in DLBCL.

**Table 3:** Research progress on ferroptosis in diffuse large B-cell lymphoma.

Researcher	Research object	Related Pathway	Target	Conclusions/ Results
Anja Schmitt (129)	GCB and ABC DLBCL cells	Lipid peroxidation	LOX/GSH/GPX4	Lipid peroxidation through the synergistic effect of high lipoxygenase expression and low levels of glutathione and glutathione peroxidase 4 in DLBCL due to DMF
Yan Zhang (130)	A DLBCL xenograft model (mice)	GSH synthesis pathway	System Xc^-^	Inhibition of the system Xc^-^ and depletion of glutathione led to lipid peroxidation in DLBCL due to imidazole ketone erastin (IKE)
Yuko Kinowaki (131)	Cases of diffuse large B-cell lymphoma (patients)	Lipid peroxidation	GPX4	GPX4 knockdown induced cell lipid peroxidation in DLBCL
Wan Seok Yang (132)	Xenograft mouse tumor models/177 cancer cell lines	Lipid peroxidation	GPX4	In DLBLC, glutathione depletion resulted in the inactivation of glutathione peroxidase (GPXs) (overexpression and downregulation of GPX4)

DLBCL can be divided into two main subtypes of germinal center B cell-like (GCB) and aggressive activated B cell-like (ABC), both of which has their own specific gene expression profile and mutation pattern. Schmitt et al. (129) investigated the antitumor mechanism of dimethyl fumarate (DMF) against DLBCL. Corresponding results revealed that DMF had a broad antitumor effect on both DLBCL subtypes, which is mediated by the induction of ferroptosis. Through the synergistic effect of high arachidate 5-lipoxygenase expression and low levels of GSH and GPX4, DMF induces lipid peroxidation in cells, leading to ferroptosis, especially GCB DLBCL. Overall, the study of DMF offers new options for the treatment of DLBCL. On the other hand, in the clinical treatment of DLBCL, sulfasalazine (SAS), a ferroptosis inducer, can inhibit GSH synthesis by suppressing SLC7A11 transport, suggesting the important role of ferroptosis in DLBCL (133). In addition, erastin and RSL3 promoted the generation of lipid ROS and induced ferroptosis in SU-DHL-8 and WSU-SLCL-2, two DLBCL cell lines, while the use of antioxidant vitamin E inhibited the progression (134).

In addition to these *in vitro* studies as mentioned above, recent clinical research (131) has precisely shown that the expression rate of GPX4 was 35.5% (33/93) in DLBCL patients, and the overall survival and progression-free survival of the GPX4-positive group were worse than those of the GPX4-negative group. It can be explained by the reason that GPX4 can reduce the intracellular lipid peroxidation level and reduce the sensitivity of cells to ferroptosis. Collectively, it suggests that increasing intracellular ROS accumulation by regulating GPX4 and system Xc^-^ multiple pathways can increase the sensitivity of lymphoma cells to ferroptosis. It may provide new promising research direction for the selection of drugs for clinical treatment of hematologic tumors.

#### 3.2.2 Burkitt’s Lymphoma

Burkitt’s lymphoma (BL) is a highly invasive B-cell malignancy, including three subsets of endemic, sporadic, and immunodeficiency-associated BL (135). Of these three variants, the specific features in endemic BL is commonly associated with the presence of Epstein–Barr virus (EBV). Currently, there are limited therapeutic options for BL patients older than 60 years old, highlighting the need for the investigation of novel treatment regimens. Artemisinin (136–138) has been identified as a new effective growth inhibitor for BL. Wang et al. (139) investigated the effect of artesunate on gene expression and its inhibitory role in BL cells DAUDI and CA-46. The results showed that artesunate induced a stress response in the endoplasmic reticulum, activation of the ATF4-CHOP-CHAC1 pathway, and degradation of intracellular GSH, ultimately reducing the resistance of lymphoma cells to ferroptosis and leading to ferroptosis in BL cells. This effect can be proven by the protective effect of LIP-1, FER-1, and DFO on cells. In addition, artemisinin inhibited CA-46 cell proliferation *in vivo* and induced ferroptosis in a mouse transplanted tumor model. Findings in this study may provide additional reference for the development of drugs targeting different types of BL.

In addition, *C-Myc* [MYC] is one of the most common inhibitory transcription factors in cancer (140–142). Its overexpression interferes with many signaling pathways to produce effect on cell growth and proliferation. Chromosomal translocations of MYC with the immunoglobulin locus were first identified in BL, resulting in abnormal structural expression of MYC, constituting the basis for establishing the *Eμ-Myc* mouse lymphoma model (143, 144). *Eμ-Myc* lymphoma mainly occurs after the acquisition of the secondary mutations, including those that inhibit tumor suppressor genes, such as p53 and ADP-ribosylation factor (ARF) (145–147). An existing research introduced the DNA sequence of *Eμ-Myc* into mice. The transgenic *Eμ-Myc* was found to be only expressed in B lymphocytes, which could cause B lymphocytes to proliferate faster than normal cells, and eventually drove the occurrence of aggressive B-cell lymphoma (148).

ALOX12 gene resides on human chromosome 17p13.1, close to that of TP53. Chu et al. (68) observed that in p53^3KR^ H1299 cells with six lipoxygenase subtypes missing, cells were treated by tert-Butyl hydroperoxide (TBH) treatment for the detection of ROS-induced ferroptosis levels. It was found that loss of function of ALOX12 specifically blocked p53-mediated ferroptosis. SLC7A11 can inhibit its enzyme activity by specifically binding to ALOX12, indicating that p53 can indirectly activate ALOX12 lipoxygenase activity by inhibiting SLC7A11 transcription and thus inhibiting the system Xc^-^, leading to ROS-induced ALOX12-dependent ferroptosis. Therefore, p53 can regulate the ferroptosis level by regulating the transcription level and activity of SLC7A11. Furthermore, in xenograft tumor models of *Eμ-Myc* lymphoma, the loss of one TP53 allele significantly accelerated *Myc*-induced classical *Eμ-Myc* lymphoma formation; however, the loss of one ALOX12 allele suppressed p53-mediated ferroptosis and eliminated p53-dependent tumor growth inhibition. It suggests that ALOX12 plays a critical role in p53-mediated ferroptosis. In addition, malignant mutation in ALOX12 gene can deprive the ability to oxidize PUFAs and induce p53-mediated ferroptosis in human tumor cells. Therefore, this study confirms that an ALOX12-mediated ferroptosis pathway is critical for p53-dependent tumor suppression, highlighting its valuable effect on the occurrence and prognosis of *Eμ-Myc* lymphoma by affecting p53 function.

### 3.3 Multiple Myeloma

MM is one of the incurable hematologic malignancies characterized by abnormal proliferation of PC at multiple sites in bone marrow, resulting in a range of tissue and organ damage (149, 150). Its clinical features include elevated serum monoclonal immunoglobulin, osteolytic destruction, and anemia, accompanied by bone marrow infiltration. In the United States, MM accounts for 1.8% of all cancers, mostly in older adults (151). Currently, bortezomib-based chemotherapy is the primary treatment regimen for MM, which can significantly prolong the survival of MM patients.

As mentioned previously, iron is an essential nutrient, which may accelerate the growth of tumor cells. Meanwhile, excess iron is also toxic, since it catalyzes the formation of ROS (152). Bordini et al. (153) reported in their study of MM model that PC may be highly sensitive to excessive iron through the production of antibodies and the synthesis of high levels of H_2_O_2_ and other by-products and eventually stimulating their production of ROS through the Fenton reaction (154, 155). Thus, inducing iron excess may inhibit the proliferation of malignant PC and enhance the effect of bortezomib, thereby controlling the progression of the disease. Through additional *in vitro* experiments, Bordini et al. (153) also cultured different MM cell lines (MMCL) *in vitro* in the presence of high doses of ferrous ammonium citrate (FeAC), with untreated and non-MM cell lines as controls. It was observed with a reduced trend of proliferation in all cell lines. In addition, iron also promoted cell death in all MMCL but not in control cells. Excess iron can be transferred out of cells by increasing FT and TF and decreasing TFR1 or CD71. For instance, PC in normal mouse expressed low TFR1 and high iron transporters to maintain low intracellular iron (156).

Indeed, MYC overexpression (157) is frequently detected in MM patients, resulting in high expression of TFR1 (153, 158), while maintaining high iron transporter levels, at least during growth in the BM microenvironment, to ensure iron excess output. The development of MM PC requires a balance between increasing iron intake to promote proliferation and avoiding iron toxicity. In a word, the accumulation of iron and ROS in MM cells may be critical in explaining the mechanism of ferroptosis, and the regulation of iron content will eventually alter the sensitivity of MM cells to ferroptosis by affecting intracellular ROS homeostasis. This study provides an effective therapeutic idea to design novel combination strategies including iron supplementation to increase the efficacy of current MM therapies.

Apart from that, Adham et al. (159) reported that *Thymus vulgaris* and *Arctium lappa* could be considered potential herbal candidates to suppress MM cell growth by promoting the production of lipid ROS and the destruction of MM PC integrity. Similarly, Zhong et al. (160) found that FTY720, a novel immunosuppressive agent, could activate AMPK subunit α (AMPKα) by activating protein phosphatase 2A (PP2A) and reducing the expression of phosphorylated eukaryotic elongation factor 2 (eEF2), which could de-phosphorylate AMP at the Thr172 site, ultimately leading to MM cell death. In 2020, Bordini et al. (161) reported that iron excess would cause the death of MM cells by lipid peroxidation. Once again, these findings strongly provide fresh perspective for the treatment of MM.

## 4 Discussion

Since its definition for the first time in 2012, ferroptosis has become an attractive target in the field of tumor research in recent decades, with much attention paid to its pathogenesis and clinical roles, providing some new ideas for tumor treatment. Ferroptosis is a novel mode of RCD induced by small-molecule compounds such as erastin and RSL3, which are regulated by multiple pathways such as iron metabolism, lipid metabolism, and GSH metabolism, as stated above. Its occurrence and development are accompanied by the lethal accumulation of ROS, leading to lipid peroxidation of cell membrane. However, there are still no specific markers of ferroptosis, and its specific mechanisms need to be further studied. The study of cell death patterns and drug resistance of tumor cells is still an important link to remove obstacles for improving the efficacy of tumor therapy. Moreover, it remains to be clarified with respect to the mechanism of ferroptosis and its relationship with diseases. For example, are there other pathways of ferroptosis regulation other than the classical pathway? Is iron essential to catalyze the generation of lipid peroxides? Or can other elements replace the role of iron in ferroptosis? Moreover, how can the results of basic research on ferroptosis be applied to clinical practice for treatment? These are the questions that need to be addressed.

Emerging studies on ferroptosis in recent decades has provided some new ideas on tumor treatment. As stated above, there is no doubt that ferroptosis plays a key role in the progression and toxicity of hematological malignancies, such as leukemia, lymphoma, and MM. Moreover, the sensitivity of hematological tumor cells to ferroptosis can be increased by regulating the level of ferroptosis-inducible factors, the balance of intracellular ROS production and extinction, and the regulation of iron metabolism homeostasis, so as to achieve the effect of killing tumor cells. In other respects, several compounds also exhibit intimate associations with ferroptosis in hematological tumor cells, and ferroptosis-inducible factor levels are correlated with the prognosis of hematological tumors. Simultaneously, the process of ferroptosis may also be affected by changes in the research progress and treatment of hematologic tumor diseases. Nevertheless, existing research on ferroptosis in hematological malignancies is still in the early stage. Further *in vivo* and *in vitro* experiments are needed to verify the effect and mechanism of ferroptosis on hematological tumor cells, which is also the direction of future research.

In summary, a comprehensive elaboration on ferroptosis and its association with hematological malignancies may benefit the understanding of the pathogenesis of these diseases and the development of highly targeted therapies with higher efficacy, despite multiple unanswered questions in this field. Effective therapeutic strategies are still being explored for hematological malignancies. Review in our study emphasizes the significance of promoting ferroptosis in the treatment of hematological malignancies. It is firmly believed that continuous research related to ferroptosis may provide further insights into this topic.

## Author Contributions

All authors contributed to the concept and design of the study. Literature collection and collation were completed by YZ and ZH. The article was written by YZ and was partially revised by ZH. All authors contributed to the article and approved the submitted version.

## Funding

This work was supported by the National Natural Science Foundation of China (82070175) and Scientific Program of Health Commission of Hunan Province (20201179).

## Conflict of Interest

The authors declare that the research was conducted in the absence of any commercial or financial relationships that could be construed as a potential conflict of interest.

## Publisher’s Note

All claims expressed in this article are solely those of the authors and do not necessarily represent those of their affiliated organizations, or those of the publisher, the editors and the reviewers. Any product that may be evaluated in this article, or claim that may be made by its manufacturer, is not guaranteed or endorsed by the publisher.

## Abbreviations

ROS, reactive oxygen species; GSH, glutathione; GPX4, glutathione peroxidase 4; RSL3, Ras-selective lethal 3; SLC7A11, solute carrier family 7 member 11; SLC3A2, solute carrier family 3 member 2; PUFA, polyunsaturated fatty acid; PL, phospholipid; IREB2, iron response element-binding protein 2; HSPB1, heat shock protein β-1; LOOH, lipid hydroperoxide; CoQ10, coenzyme Q10; SFA, saturated fatty acid; MUFA, monounsaturated fatty acid; AA, arachidonic acid; ADA, adrenic acid; PE, phosphatidylethanolamine; ACSL4, acyl-CoA synthetase long-chain family member 4; LPCAT3, lysophosphatidylcholine acyltransferase 3; LOX, lipoxygenase; H_2_O_2_, hydrogen peroxide; Fer-1, ferrostatin-1; Lip-1, liproxstatin-1; DFO, deferoxamine; TF, transferrin; TFR1, transferrin receptor 1; DMT1, divalent metal transporter 1; LIP, labile iron pool; FT, ferritin; FTH1, ferritin heavy chain 1; FTL, ferritin light chain; FSP1, ferroptosis suppressor protein 1; NCOA4, nuclear receptor coactivator 4; FPN, ferroportin; Se, selenium; Sec, selenocysteine; L-OH, lipid alcohol; Sec, selenocysteine; IPP, isopentenyl pyrophosphate; HMG-CoA, 3-hydroxy-3-methyl glutaryl-coenzyme A; HMGCR, HMG-CoA reductase; FSP1, ferroptosis suppressor protein 1; NRF2, nuclear factor erythroid 2-related factor 2; p62, nucleoporin 62; NOX, NADPH oxidase; DPI, diphenylene iodonium; TCA, cycle tricarboxylic acid cycle; Keap1, Kelch-like ECH-associated protein; HO-1, heme oxygenase-1; IRP, iron regulatory protein; System Xc-, cystine/glutamate reverse transporter.

## References

[1] Fink SL Cookson BT. Apoptosis, Pyroptosis, and Necrosis: Mechanistic Description of Dead and Dying Eukaryotic Cells. Infect Immun (2005) 73(4):1907–16. doi: 10.1128/iai.73.4.1907-1916.2005 PMC1087413 15784530

[2] Dolma S Lessnick SL Hahn WC Stockwell BR. Identification of Genotype-Selective Antitumor Agents Using Synthetic Lethal Chemical Screening in Engineered Human Tumor Cells. Cancer Cell (2003) 3(3):285–96. doi: 10.1016/s1535-6108(03)00050-3 12676586

[3] Yang WS Stockwell BR. Synthetic Lethal Screening Identifies Compounds Activating Iron-Dependent, Nonapoptotic Cell Death in Oncogenic-RAS-Harboring Cancer Cells. Chem Biol (2008) 15(3):234–45. doi: 10.1016/j.chembiol.2008.02.010 PMC2683762 18355723

[4] Dixon SJ Lemberg KM Lamprecht MR Skouta R Zaitsev EM Gleason CE. Ferroptosis: An Iron-Dependent Form of Nonapoptotic Cell Death. Cell (2012) 149(5):1060–72. doi: 10.1016/j.cell.2012.03.042 PMC3367386 22632970

[5] Xie Y Hou W Song X Yu Y Huang J Sun X. Ferroptosis: Process and Function. Cell Death Differ (2016) 23(3):369–79. doi: 10.1038/cdd.2015.158 PMC5072448 26794443

[6] Friedmann Angeli JP Schneider M Proneth B Tyurina YY Tyurin VA Hammond VJ. Inactivation of the Ferroptosis Regulator Gpx4 Triggers Acute Renal Failure in Mice. Nat Cell Biol (2014) 16(12):1180–91. doi: 10.1038/ncb3064 PMC4894846 25402683

[7] Stockwell BR Friedmann Angeli JP Bayir H Bush AI Conrad M Dixon SJ. Ferroptosis: A Regulated Cell Death Nexus Linking Metabolism, Redox Biology, and Disease. Cell (2017) 171(2):273–85. doi: 10.1016/j.cell.2017.09.021 PMC5685180 28985560

[8] Agmon E Solon J Bassereau P Stockwell BR. Modeling the Effects of Lipid Peroxidation During Ferroptosis on Membrane Properties. Sci Rep (2018) 8(1):5155. doi: 10.1038/s41598-018-23408-0 29581451 PMC5979948

[9] Li J Cao F Yin HL Huang ZJ Lin ZT Mao N. Ferroptosis: Past, Present and Future. Cell Death Dis (2020) 11(2):88. doi: 10.1038/s41419-020-2298-2 32015325 PMC6997353

[10] Ooko E Saeed ME Kadioglu O Sarvi S Colak M Elmasaoudi K. Artemisinin Derivatives Induce Iron-Dependent Cell Death (Ferroptosis) in Tumor Cells. Phytomedicine (2015) 22(11):1045–54. doi: 10.1016/j.phymed.2015.08.002 26407947

[11] Hassan W Noreen H Khalil S Hussain A Rehman S Sajjad S. Ethanolic Extract of Nigella Sativa Protects Fe(II) Induced Lipid Peroxidation in Rat’s Brain, Kidney and Liver Homogenates. Pak J Pharm Sci (2016) 29(1):231–7. 26826815

[12] Tang D Kroemer G. Ferroptosis. Curr Biol (2020) 30(21):R1292–7. doi: 10.1016/j.cub.2020.09.068 33142092

[13] Chen X Li J Kang R Klionsky DJ Tang D. Ferroptosis: Machinery and Regulation. Autophagy (2020) 17(9):2054–81. doi: 10.1080/15548627.2020.1810918 PMC8496712 32804006

[14] Gao M Monian P Pan Q Zhang W Xiang J Jiang X. Ferroptosis is an Autophagic Cell Death Process. Cell Res (2016) 26(9):1021–32. doi: 10.1038/cr.2016.95 PMC5034113 27514700

[15] Gao M Monian P Quadri N Ramasamy R Jiang X. Glutaminolysis and Transferrin Regulate Ferroptosis. Mol Cell (2015) 59(2):298–308. doi: 10.1016/j.molcel.2015.06.011 26166707 PMC4506736

[16] Zou Y Palte MJ Deik AA Li H Eaton JK Wang W. A GPX4-Dependent Cancer Cell State Underlies the Clear-Cell Morphology and Confers Sensitivity to Ferroptosis. Nat Commun (2019) 10(1):1617. doi: 10.1038/s41467-019-09277-9 30962421 PMC6453886

[17] Kazan HH Urfali-Mamatoglu C Gunduz U. Iron Metabolism and Drug Resistance in Cancer. Biometals (2017) 30(5):629–41. doi: 10.1007/s10534-017-0037-7 28766192

[18] Gao M Monian P Jiang X. Metabolism and Iron Signaling in Ferroptotic Cell Death. Oncotarget (2015) 6(34):35145–6. doi: 10.18632/oncotarget.5671 PMC4742090 26387139

[19] Sun X Ou Z Xie M Kang R Fan Y Niu X. HSPB1 as a Novel Regulator of Ferroptotic Cancer Cell Death. Oncogene (2015) 34(45):5617–25. doi: 10.1038/onc.2015.32 PMC4640181 25728673

[20] Hamaï A Mehrpour M. [Autophagy and Iron Homeostasis]. Med Sci (Paris) (2017) 33(3):260–7. doi: 10.1051/medsci/20173303012 28367812

[21] Pantopoulos K Porwal SK Tartakoff A Devireddy L. Mechanisms of Mammalian Iron Homeostasis. Biochemistry (2012) 51(29):5705–24. doi: 10.1021/bi300752r PMC3572738 22703180

[22] Kwon MY Park E Lee SJ Chung SW. Heme Oxygenase-1 Accelerates Erastin-Induced Ferroptotic Cell Death. Oncotarget (2015) 6(27):24393–403. doi: 10.18632/oncotarget.5162 PMC4695193 26405158

[23] Harrison PM Arosio P. The Ferritins: Molecular Properties, Iron Storage Function and Cellular Regulation. Biochim Biophys Acta (1996) 1275(3):161–203. doi: 10.1016/0005-2728(96)00022-9 8695634

[24] Gammella E Recalcati S Rybinska I Buratti P Cairo G. Iron-Induced Damage in Cardiomyopathy: Oxidative-Dependent and Independent Mechanisms. Oxid Med Cell Longev (2015) 2015:230182. doi: 10.1155/2015/230182 25878762 PMC4387903

[25] Latunde-Dada GO. Ferroptosis: Role of Lipid Peroxidation, Iron and Ferritinophagy. Biochim Biophys Acta Gen Subj (2017) 1861(8):1893–900. doi: 10.1016/j.bbagen.2017.05.019 28552631

[26] Kang R Tang D. Autophagy and Ferroptosis - What’s the Connection? Curr Pathobiol Rep (2017) 5(2):153–9. doi: 10.1007/s40139-017-0139-5 PMC5640172 29038744

[27] Mancias JD Wang X Gygi SP Harper JW Kimmelman AC. Quantitative Proteomics Identifies NCOA4 as the Cargo Receptor Mediating Ferritinophagy. Nature (2014) 509(7498):105–9. doi: 10.1038/nature13148 PMC4180099 24695223

[28] Hou W Xie Y Song X Sun X Lotze MT Zeh HJ. 3rd Autophagy Promotes Ferroptosis by Degradation of Ferritin. Autophagy (2016) 12(8):1425–8. doi: 10.1080/15548627.2016.1187366 PMC4968231 27245739

[29] Strzyz P. Iron Expulsion by Exosomes Drives Ferroptosis Resistance. Nat Rev Mol Cell Biol (2020) 21(1):4–5. doi: 10.1038/s41580-019-0195-2 31748716

[30] Brown CW Chhoy P Mukhopadhyay D Karner ER Mercurio AM. Targeting Prominin2 Transcription to Overcome Ferroptosis Resistance in Cancer. EMBO Mol Med (2021) 13(8):e13792. doi: 10.15252/emmm.202013792 34223704 PMC8350900

[31] Brown CW Amante JJ Chhoy P Elaimy AL Liu H Zhu LJ. Prominin2 Drives Ferroptosis Resistance by Stimulating Iron Export. Dev Cell (2019) 51(5):575–86.e4. doi: 10.1016/j.devcel.2019.10.007 31735663 PMC8316835

[32] Bogdan AR Miyazawa M Hashimoto K Tsuji Y. Regulators of Iron Homeostasis: New Players in Metabolism, Cell Death, and Disease. Trends Biochem Sci (2016) 41(3):274–86. doi: 10.1016/j.tibs.2015.11.012 PMC4783254 26725301

[33] Anderson CP Shen M Eisenstein RS Leibold EA. Mammalian Iron Metabolism and its Control by Iron Regulatory Proteins. Biochim Biophys Acta (2012) 1823(9):1468–83. doi: 10.1016/j.bbamcr.2012.05.010 PMC3675657 22610083

[34] Hassannia B Vandenabeele P Vanden Berghe T. Targeting Ferroptosis to Iron Out Cancer. Cancer Cell (2019) 35(6):830–49. doi: 10.1016/j.ccell.2019.04.002 31105042

[35] Cheng Z Li Y. What is Responsible for the Initiating Chemistry of Iron-Mediated Lipid Peroxidation: An Update. Chem Rev (2007) 107(3):748–66. doi: 10.1021/cr040077w 17326688

[36] Su LJ Zhang JH Gomez H Murugan R Hong X Xu D. Reactive Oxygen Species-Induced Lipid Peroxidation in Apoptosis, Autophagy, and Ferroptosis. Oxid Med Cell Longev (2019) 2019:5080843. doi: 10.1155/2019/5080843 31737171 PMC6815535

[37] Viswanathan VS Ryan MJ Dhruv HD Gill S Eichhoff OM Seashore-Ludlow B. Dependency of a Therapy-Resistant State of Cancer Cells on a Lipid Peroxidase Pathway. Nature (2017) 547(7664):453–7. doi: 10.1038/nature23007 PMC5667900 28678785

[38] Lundgren CAK Sjöstrand D Biner O Bennett M Rudling A Johansson AL. Scavenging of Superoxide by a Membrane-Bound Superoxide Oxidase. Nat Chem Biol (2018) 14(8):788–93. doi: 10.1038/s41589-018-0072-x PMC6379055 29915379

[39] Chen X Kang R Kroemer G Tang D. Broadening Horizons: The Role of Ferroptosis in Cancer. Nat Rev Clin Oncol (2021) 18(5):280–96. doi: 10.1038/s41571-020-00462-0 33514910

[40] Doll S Proneth B Tyurina YY Panzilius E Kobayashi S Ingold I. ACSL4 Dictates Ferroptosis Sensitivity by Shaping Cellular Lipid Composition. Nat Chem Biol (2017) 13(1):91–8. doi: 10.1038/nchembio.2239 PMC5610546 27842070

[41] Kagan VE Mao G Qu F Angeli JP Doll S Croix CS. Oxidized Arachidonic and Adrenic PEs Navigate Cells to Ferroptosis. Nat Chem Biol (2017) 13(1):81–90. doi: 10.1038/nchembio.2238 27842066 PMC5506843

[42] Magtanong L Dixon SJ. Ferroptosis and Brain Injury. Dev Neurosci (2018) 40(5-6):382–95. doi: 10.1159/000496922 PMC6658337 30820017

[43] Kuhn H Banthiya S van Leyen K. Mammalian Lipoxygenases and Their Biological Relevance. Biochim Biophys Acta (2015) 1851(4):308–30. doi: 10.1016/j.bbalip.2014.10.002 PMC4370320 25316652

[44] Wenzel SE Tyurina YY Zhao J St Croix CM Dar HH Mao G. PEBP1 Wardens Ferroptosis by Enabling Lipoxygenase Generation of Lipid Death Signals. Cell (2017) 171(3):628–41.e26. doi: 10.1016/j.cell.2017.09.044 29053969 PMC5683852

[45] Seiler A Schneider M Förster H Roth S Wirth EK Culmsee C. Glutathione Peroxidase 4 Senses and Translates Oxidative Stress Into 12/15-Lipoxygenase Dependent- and AIF-Mediated Cell Death. Cell Metab (2008) 8(3):237–48. doi: 10.1016/j.cmet.2008.07.005 18762024

[46] Yang WS SriRamaratnam R Welsch ME Shimada K Skouta R Viswanathan VS. Regulation of Ferroptotic Cancer Cell Death by GPX4. Cell (2014) 156(1-2):317–31. doi: 10.1016/j.cell.2013.12.010 PMC4076414 24439385

[47] Seibt TM Proneth B Conrad M. Role of GPX4 in Ferroptosis and its Pharmacological Implication. Free Radic Biol Med (2019) 133:144–52. doi: 10.1016/j.freeradbiomed.2018.09.014 30219704

[48] Yang WS Stockwell BR. Ferroptosis: Death by Lipid Peroxidation. Trends Cell Biol (2016) 26(3):165–76. doi: 10.1016/j.tcb.2015.10.014 PMC4764384 26653790

[49] Friedmann Angeli JP Conrad M. Selenium and GPX4, a Vital Symbiosis. Free Radic Biol Med (2018) 127:153–9. doi: 10.1016/j.freeradbiomed.2018.03.001 29522794

[50] Cardoso BR Hare DJ Bush AI Roberts BR. Glutathione Peroxidase 4: A New Player in Neurodegeneration? Mol Psychiatry (2017) 22(3):328–35. doi: 10.1038/mp.2016.196 27777421

[51] Alim I Caulfield JT Chen Y Swarup V Geschwind DH Ivanova E. Selenium Drives a Transcriptional Adaptive Program to Block Ferroptosis and Treat Stroke. Cell (2019) 177(5):1262–79.e25. doi: 10.1016/j.cell.2019.03.032 31056284

[52] Ingold I Berndt C Schmitt S Doll S Poschmann G Buday K. Selenium Utilization by GPX4 Is Required to Prevent Hydroperoxide-Induced Ferroptosis. Cell (2018) 172(3):409–22.e21. doi: 10.1016/j.cell.2017.11.048 29290465

[53] Gaschler MM Andia AA Liu H Csuka JM Hurlocker B Vaiana CA. FINO(2) Initiates Ferroptosis Through GPX4 Inactivation and Iron Oxidation. Nat Chem Biol (2018) 14(5):507–15. doi: 10.1038/s41589-018-0031-6 PMC5899674 29610484

[54] Shimada K Skouta R Kaplan A Yang WS Hayano M Dixon SJ. Global Survey of Cell Death Mechanisms Reveals Metabolic Regulation of Ferroptosis. Nat Chem Biol (2016) 12(7):497–503. doi: 10.1038/nchembio.2079 27159577 PMC4920070

[55] Gao M Jiang X. To Eat or Not to Eat-The Metabolic Flavor of Ferroptosis. Curr Opin Cell Biol (2018) 51:58–64. doi: 10.1016/j.ceb.2017.11.001 29175614 PMC5949249

[56] Xie B Guo Y. Molecular Mechanism of Cell Ferroptosis and Research Progress in Regulation of Ferroptosis by Noncoding RNAs in Tumor Cells. Cell Death Discovery (2021) 7(1):101. doi: 10.1038/s41420-021-00483-3 33980834 PMC8115351

[57] Sato H Tamba M Ishii T Bannai S. Cloning and Expression of a Plasma Membrane Cystine/Glutamate Exchange Transporter Composed of Two Distinct Proteins. J Biol Chem (1999) 274(17):11455–8. doi: 10.1074/jbc.274.17.11455 10206947

[58] Koppula P Zhang Y Zhuang L Gan B. Amino Acid Transporter SLC7A11/xCT at the Crossroads of Regulating Redox Homeostasis and Nutrient Dependency of Cancer. Cancer Commun (Lond) (2018) 38(1):12. doi: 10.1186/s40880-018-0288-x 29764521 PMC5993148

[59] Imai H Matsuoka M Kumagai T Sakamoto T Koumura T. Lipid Peroxidation-Dependent Cell Death Regulated by GPx4 and Ferroptosis. Curr Top Microbiol Immunol (2017) 403:143–70. doi: 10.1007/82_2016_508 28204974

[60] Conrad M Sato H. The Oxidative Stress-Inducible Cystine/Glutamate Antiporter, System X (C) (-): Cystine Supplier and Beyond. Amino Acids (2012) 42(1):231–46. doi: 10.1007/s00726-011-0867-5 21409388

[61] Tan Q Fang Y Gu Q. Mechanisms of Modulation of Ferroptosis and Its Role in Central Nervous System Diseases. Front Pharmacol (2021) 12:657033. doi: 10.3389/fphar.2021.657033 34149412 PMC8213017

[62] Maiorino M Conrad M Ursini F. GPx4, Lipid Peroxidation, and Cell Death: Discoveries, Rediscoveries, and Open Issues. Antioxid Redox Signal (2018) 29(1):61–74. doi: 10.1089/ars.2017.7115 28462584

[63] Ursini F Maiorino M. Lipid Peroxidation and Ferroptosis: The Role of GSH and Gpx4. Free Radic Biol Med (2020) 152:175–85. doi: 10.1016/j.freeradbiomed.2020.02.027 32165281

[64] Jiang L Hickman JH Wang SJ Gu W. Dynamic Roles of P53-Mediated Metabolic Activities in ROS-Induced Stress Responses. Cell Cycle (2015) 14(18):2881–5. doi: 10.1080/15384101.2015.1068479 PMC4825584 26218928

[65] Jiang L Kon N Li T Wang SJ Su T Hibshoosh H. Ferroptosis as a P53-Mediated Activity During Tumour Suppression. Nature (2015) 520(7545):57–62. doi: 10.1038/nature14344 25799988 PMC4455927

[66] Chen D Chu B Yang X Liu Z Jin Y Kon N. Ipla2β-Mediated Lipid Detoxification Controls P53-Driven Ferroptosis Independent of GPX4. Nat Commun (2021) 12(1):3644. doi: 10.1038/s41467-021-23902-6 34131139 PMC8206155

[67] Tarangelo A Magtanong L Bieging-Rolett KT Li Y Ye J Attardi LD. P53 Suppresses Metabolic Stress-Induced Ferroptosis in Cancer Cells. Cell Rep (2018) 22(3):569–75. doi: 10.1016/j.celrep.2017.12.077 PMC5791910 29346757

[68] Chu B Kon N Chen D Li T Liu T Jiang L. ALOX12 Is Required for P53-Mediated Tumour Suppression Through a Distinct Ferroptosis Pathway. Nat Cell Biol (2019) 21(5):579–91. doi: 10.1038/s41556-019-0305-6 PMC6624840 30962574

[69] Liu Y Chen C Xu Z Scuoppo C Rillahan CD Gao J. Deletions Linked to TP53 Loss Drive Cancer Through P53-Independent Mechanisms. Nature (2016) 531(7595):471–5. doi: 10.1038/nature17157 PMC4836395 26982726

[70] Bersuker K Hendricks JM Li Z Magtanong L Ford B Tang PH. The CoQ Oxidoreductase FSP1 Acts Parallel to GPX4 to Inhibit Ferroptosis. Nature (2019) 575(7784):688–92. doi: 10.1038/s41586-019-1705-2 PMC6883167 31634900

[71] Doll S Freitas FP Shah R Aldrovandi M da Silva MC Ingold I. FSP1 is a Glutathione-Independent Ferroptosis Suppressor. Nature (2019) 575(7784):693–8. doi: 10.1038/s41586-019-1707-0 31634899

[72] Shimada K Hayano M Pagano NC Stockwell BR. Cell-Line Selectivity Improves the Predictive Power of Pharmacogenomic Analyses and Helps Identify NADPH as Biomarker for Ferroptosis Sensitivity. Cell Chem Biol (2016) 23(2):225–35. doi: 10.1016/j.chembiol.2015.11.016 PMC4792701 26853626

[73] Xie Y Zhu S Song X Sun X Fan Y Liu J. The Tumor Suppressor P53 Limits Ferroptosis by Blocking DPP4 Activity. Cell Rep (2017) 20(7):1692–704. doi: 10.1016/j.celrep.2017.07.055 28813679

[74] Shiose A Sumimoto H. Arachidonic Acid and Phosphorylation Synergistically Induce a Conformational Change of P47phox to Activate the Phagocyte NADPH Oxidase. J Biol Chem (2000) 275(18):13793–801. doi: 10.1074/jbc.275.18.13793 10788501

[75] Calses PC Crawford JJ Lill JR Dey A. Hippo Pathway in Cancer: Aberrant Regulation and Therapeutic Opportunities. Trends Cancer (2019) 5(5):297–307. doi: 10.1016/j.trecan.2019.04.001 31174842

[76] Meng Z Moroishi T Guan KL. Mechanisms of Hippo Pathway Regulation. Genes Dev (2016) 30(1):1–17. doi: 10.1101/gad.274027.115 26728553 PMC4701972

[77] Nguyen CDK Yi C. YAP/TAZ Signaling and Resistance to Cancer Therapy. Trends Cancer (2019) 5(5):283–96. doi: 10.1016/j.trecan.2019.02.010 PMC6557283 31174841

[78] Ma S Meng Z Chen R Guan KL. The Hippo Pathway: Biology and Pathophysiology. Annu Rev Biochem (2019) 88:577–604. doi: 10.1146/annurev-biochem-013118-111829 30566373

[79] Luo M Wu L Zhang K Wang H Zhang T Gutierrez L. miR-137 Regulates Ferroptosis by Targeting Glutamine Transporter SLC1A5 in Melanoma. Cell Death Differ (2018) 25(8):1457–72. doi: 10.1038/s41418-017-0053-8 PMC6113319 29348676

[80] Mao C Liu X Zhang Y Lei G Yan Y Lee H. DHODH-Mediated Ferroptosis Defence Is a Targetable Vulnerability in Cancer. Nature (2021) 593(7860):586–90. doi: 10.1038/s41586-021-03539-7 PMC8895686 33981038

[81] Derry PJ Hegde ML Jackson GR Kayed R Tour JM Tsai AL. Revisiting the Intersection of Amyloid, Pathologically Modified Tau and Iron in Alzheimer’s Disease From a Ferroptosis Perspective. Prog Neurobiol (2020) 184:101716. doi: 10.1016/j.pneurobio.2019.101716 31604111 PMC7850812

[82] Louandre C Marcq I Bouhlal H Lachaier E Godin C Saidak Z. The Retinoblastoma (Rb) Protein Regulates Ferroptosis Induced by Sorafenib in Human Hepatocellular Carcinoma Cells. Cancer Lett (2015) 356(2 Pt B):971–7. doi: 10.1016/j.canlet.2014.11.014 25444922

[83] Hao S Yu J He W Huang Q Zhao Y Liang B. Cysteine Dioxygenase 1 Mediates Erastin-Induced Ferroptosis in Human Gastric Cancer Cells. Neoplasia (2017) 19(12):1022–32. doi: 10.1016/j.neo.2017.10.005 PMC5686465 29144989

[84] Alvarez SW Sviderskiy VO Terzi EM Papagiannakopoulos T Moreira AL Adams S. NFS1 Undergoes Positive Selection in Lung Tumours and Protects Cells From Ferroptosis. Nature (2017) 551(7682):639–43. doi: 10.1038/nature24637 PMC5808442 29168506

[85] Ma D Li C Jiang P Jiang Y Wang J Zhang D. Inhibition of Ferroptosis Attenuates Acute Kidney Injury in Rats With Severe Acute Pancreatitis. Dig Dis Sci (2021) 66(2):483–92. doi: 10.1007/s10620-020-06225-2 32219613

[86] Dai C Li H Wang Y Tang S Velkov T Shen J. Inhibition of Oxidative Stress and ALOX12 and NF-κb Pathways Contribute to the Protective Effect of Baicalein on Carbon Tetrachloride-Induced Acute Liver Injury. Antioxid (Basel) (2021) 10(6):976. doi: 10.3390/antiox10060976 PMC8235740 34207230

[87] Eling N Reuter L Hazin J Hamacher-Brady A Brady NR. Identification of Artesunate as a Specific Activator of Ferroptosis in Pancreatic Cancer Cells. Oncoscience (2015) 2(5):517–32. doi: 10.18632/oncoscience.160 PMC4468338 26097885

[88] Bowman RL Busque L Levine RL. Clonal Hematopoiesis and Evolution to Hematopoietic Malignancies. Cell Stem Cell (2018) 22(2):157–70. doi: 10.1016/j.stem.2018.01.011 PMC5804896 29395053

[89] Armitage JO Gascoyne RD Lunning MA Cavalli F. Non-Hodgkin Lymphoma. Lancet (2017) 390(10091):298–310. doi: 10.1016/s0140-6736(16)32407-2 28153383

[90] Gurnari C Voso MT Maciejewski JP Visconte V. From Bench to Bedside and Beyond: Therapeutic Scenario in Acute Myeloid Leukemia. Cancers (Basel) (2020) 12(2):357. doi: 10.3390/cancers12020357 PMC7072629 32033196

[91] Ziccheddu B Da Via MC Lionetti M Maeda A Morlupi S Dugo M. Functional Impact of Genomic Complexity on the Transcriptome of Multiple Myeloma. Clin Cancer Res (2021). doi: 10.1158/1078-0432.Ccr-20-4366 PMC7612071 34526359

[92] Lebon D Vergez F Bertoli S Harrivel V De Botton S Micol JB. Hyperferritinemia at Diagnosis Predicts Relapse and Overall Survival in Younger AML Patients With Intermediate-Risk Cytogenetics. Leuk Res (2015) 39(8):818–21. doi: 10.1016/j.leukres.2015.05.001 26002512

[93] Bertoli S Paubelle E Bérard E Saland E Thomas X Tavitian S. Ferritin Heavy/Light Chain (FTH1/FTL) Expression, Serum Ferritin Levels, and Their Functional as Well as Prognostic Roles in Acute Myeloid Leukemia. Eur J Haematol (2019) 102(2):131–42. doi: 10.1111/ejh.13183 30325535

[94] Gasparetto M Pei S Minhajuddin M Stevens B Smith CA Seligman P. Low Ferroportin Expression in AML is Correlated With Good Risk Cytogenetics, Improved Outcomes and Increased Sensitivity to Chemotherapy. Leuk Res (2019) 80:1–10. doi: 10.1016/j.leukres.2019.02.011 30852438

[95] Wermke M Eckoldt J Götze KS Klein SA Bug G de Wreede LC. Enhanced Labile Plasma Iron and Outcome in Acute Myeloid Leukaemia and Myelodysplastic Syndrome After Allogeneic Haemopoietic Cell Transplantation (ALLIVE): A Prospective, Multicentre, Observational Trial. Lancet Haematol (2018) 5(5):e201–10. doi: 10.1016/s2352-3026(18)30036-x 29628397

[96] Duca L Cappellini MD Baronciani D Pilo F Targhetta C Visani G. Non-Transferrin-Bound Iron and Oxidative Stress During Allogeneic Hemopoietic Stem Cell Transplantation in Patients With or Without Iron Overload. Am J Hematol (2018) 93(9):E250–2. doi: 10.1002/ajh.25201 29981284

[97] Sahlstedt L Ebeling F von Bonsdorff L Parkkinen J Ruutu T. Non-Transferrin-Bound Iron During Allogeneic Stem Cell Transplantation. Br J Haematol (2001) 113(3):836–8. doi: 10.1046/j.1365-2141.2001.02820.x 11380478

[98] Cheng PP Sun ZZ Jiang F Tang YT Jiao XY. Hepcidin Expression in Patients With Acute Leukaemia. Eur J Clin Invest (2012) 42(5):517–25. doi: 10.1111/j.1365-2362.2011.02608.x 22023453

[99] De Kouchkovsky I Abdul-Hay M. ‘Acute Myeloid Leukemia: A Comprehensive Review and 2016 Update’. Blood Cancer J (2016) 6(7):e441. doi: 10.1038/bcj.2016.50 27367478 PMC5030376

[100] Torii S Shintoku R Kubota C Yaegashi M Torii R Sasaki M. An Essential Role for Functional Lysosomes in Ferroptosis of Cancer Cells. Biochem J (2016) 473(6):769–77. doi: 10.1042/bj20150658 26759376

[101] Friedmann Angeli JP Krysko DV Conrad M. Ferroptosis at the Crossroads of Cancer-Acquired Drug Resistance and Immune Evasion. Nat Rev Cancer (2019) 19(7):405–14. doi: 10.1038/s41568-019-0149-1 31101865

[102] Shen Z Song J Yung BC Zhou Z Wu A Chen X. Emerging Strategies of Cancer Therapy Based on Ferroptosis. Adv Mater (2018) 30(12):e1704007. doi: 10.1002/adma.201704007 29356212 PMC6377162

[103] Lou S Hong H Maihesuti L Gao H Zhu Z Xu L. Inhibitory Effect of Hydnocarpin D on T-Cell Acute Lymphoblastic Leukemia *via* Induction of Autophagy-Dependent Ferroptosis. Exp Biol Med (Maywood) (2021) 246(13):1541–53. doi: 10.1177/15353702211004870 PMC8283251 33926261

[104] Probst L Dächert J Schenk B Fulda S. Lipoxygenase Inhibitors Protect Acute Lymphoblastic Leukemia Cells From Ferroptotic Cell Death. Biochem Pharmacol (2017) 140:41–52. doi: 10.1016/j.bcp.2017.06.112 28595877

[105] Dächert J Schoeneberger H Rohde K Fulda S. RSL3 and Erastin Differentially Regulate Redox Signaling to Promote Smac Mimetic-Induced Cell Death. Oncotarget (2016) 7(39):63779–92. doi: 10.18632/oncotarget.11687 PMC5325403 27588473

[106] Mbaveng AT Ndontsa BL Kuete V Nguekeu YMM Çelik İ Mbouangouere R. A Naturally Occuring Triterpene Saponin Ardisiacrispin B Displayed Cytotoxic Effects in Multi-Factorial Drug Resistant Cancer Cells *via* Ferroptotic and Apoptotic Cell Death. Phytomedicine (2018) 43:78–85. doi: 10.1016/j.phymed.2018.03.035 29747757

[107] Mbaveng AT Fotso GW Ngnintedo D Kuete V Ngadjui BT Keumedjio F. Cytotoxicity of Epunctanone and Four Other Phytochemicals Isolated From the Medicinal Plants Garcinia Epunctata and Ptycholobium Contortum Towards Multi-Factorial Drug Resistant Cancer Cells. Phytomedicine (2018) 48:112–9. doi: 10.1016/j.phymed.2017.12.016 30195869

[108] Aldoss I Marcucci G. More Options for Older Patients With Acute Myeloid Leukemia: Venetoclax in Combination With Low Dose Cytarabine. Chin Clin Oncol (2019) 8(S1):S25. doi: 10.21037/cco.2019.09.03 31684734

[109] Hu L Gao Y Shi Z Liu Y Zhao J Xiao Z. DNA Methylation-Based Prognostic Biomarkers of Acute Myeloid Leukemia Patients. Ann Transl Med (2019) 7(23):737. doi: 10.21037/atm.2019.11.122 32042753 PMC6989983

[110] Weber S Parmon A Kurrle N Schnütgen F Serve H. The Clinical Significance of Iron Overload and Iron Metabolism in Myelodysplastic Syndrome and Acute Myeloid Leukemia. Front Immunol (2020) 11:627662. doi: 10.3389/fimmu.2020.627662 33679722 PMC7933218

[111] Grignano E Birsen R Chapuis N Bouscary D. From Iron Chelation to Overload as a Therapeutic Strategy to Induce Ferroptosis in Leukemic Cells. Front Oncol (2020) 10:586530. doi: 10.3389/fonc.2020.586530 33042852 PMC7530268

[112] Birsen R Larrue C Decroocq J Johnson N Guiraud N Gotanegre M. APR-246 Induces Early Cell Death by Ferroptosis in Acute Myeloid Leukemia. Haematologica (2021). doi: 10.3324/haematol.2020.259531 PMC8804578 33406814

[113] Wei J Xie Q Liu X Wan C Wu W Fang K. Identification the Prognostic Value of Glutathione Peroxidases Expression Levels in Acute Myeloid Leukemia. Ann Transl Med (2020) 8(11):678. doi: 10.21037/atm-20-3296 32617298 PMC7327321

[114] Du Y Bao J Zhang MJ Li LL Xu XL Chen H. Targeting Ferroptosis Contributes to ATPR-Induced AML Differentiation *via* ROS-Autophagy-Lysosomal Pathway. Gene (2020) 755:144889. doi: 10.1016/j.gene.2020.144889 32534056

[115] Zhu HY Huang ZX Chen GQ Sheng F Zheng YS. Typhaneoside Prevents Acute Myeloid Leukemia (AML) Through Suppressing Proliferation and Inducing Ferroptosis Associated With Autophagy. Biochem Biophys Res Commun (2019) 516(4):1265–71. doi: 10.1016/j.bbrc.2019.06.070 31301767

[116] Yusuf RZ Saez B Sharda A van Gastel N Yu VWC Baryawno N. Aldehyde Dehydrogenase 3a2 Protects AML Cells From Oxidative Death and the Synthetic Lethality of Ferroptosis Inducers. Blood (2020) 136(11):1303–16. doi: 10.1182/blood.2019001808 PMC7483435 32458004

[117] Ye F Chai W Xie M Yang M Yu Y Cao L. HMGB1 Regulates Erastin-Induced Ferroptosis *via* RAS-JNK/p38 Signaling in HL-60/NRAS(Q61L) Cells. Am J Cancer Res (2019) 9(4):730–9. PMC6511643 31105999

[118] Du J Wang T Li Y Zhou Y Wang X Yu X. DHA Inhibits Proliferation and Induces Ferroptosis of Leukemia Cells Through Autophagy Dependent Degradation of Ferritin. Free Radic Biol Med (2019) 131:356–69. doi: 10.1016/j.freeradbiomed.2018 30557609

[119] Sagasser J Ma BN Baecker D Salcher S Hermann M Lamprecht J. A New Approach in Cancer Treatment: Discovery of Chlorido[N,N'-Disalicylidene-1,2-Phenylenediamine]iron(III) Complexes as Ferroptosis Inducers. J Med Chem (2019) 62(17):8053–61. doi: 10.1021/acs.jmedchem.9b00814 31369259

[120] Baecker D Ma BN Sagasser J Schultz L Hörschläger C Weinreich M. Amide and Ester Derivatives of Chlorido[4-Carboxy-1,2-Disalicylideneaminobenzene]iron(iii) as Necroptosis and Ferroptosis Inducers. Dalton Trans (2020) 49(20):6842–53. doi: 10.1039/d0dt00168f 32377663

[121] Yu Y Xie Y Cao L Yang L Yang M Lotze MT. The Ferroptosis Inducer Erastin Enhances Sensitivity of Acute Myeloid Leukemia Cells to Chemotherapeutic Agents. Mol Cell Oncol (2015) 2(4):e1054549. doi: 10.1080/23723556.2015.1054549 27308510 PMC4905356

[122] Dong LH Huang JJ Zu P Liu J Gao X Du JW. CircKDM4C Upregulates P53 by Sponging hsa-let-7b-5p to Induce Ferroptosis in Acute Myeloid Leukemia. Environ Toxicol (2021) 36(7):1288–302. doi: 10.1002/tox.23126 33733556

[123] Chen Z Jiang J Fu N Chen L. Targetting Ferroptosis for Blood Cell-Related Diseases. J Drug Target (2021) 1–15. doi: 10.1080/1061186x.2021.1971237 34415804

[124] Smaldone G Coppola L Pane K Franzese M Beneduce G Parasole R. KCTD15 Deregulation Is Associated With Alterations of the NF-κb Signaling in Both Pathological and Physiological Model Systems. Sci Rep (2021) 11(1):18237. doi: 10.1038/s41598-021-97775-6 34521919 PMC8440651

[125] Nilson I Löchner K Siegler G Greil J Beck JD Fey GH. Exon/intron Structure of the Human ALL-1 (MLL) Gene Involved in Translocations to Chromosomal Region 11q23 and Acute Leukaemias. Br J Haematol (1996) 93(4):966–72. doi: 10.1046/j.1365-2141.1996.d01-1748.x 8703835

[126] Saarinen-Pihkala UM Gustafsson G Carlsen N Flaegstad T Forestier E Glomstein A. Outcome of Children With High-Risk Acute Lymphoblastic Leukemia (HR-ALL): Nordic Results on an Intensive Regimen With Restricted Central Nervous System Irradiation. Pediatr Blood Cancer (2004) 42(1):8–23. doi: 10.1002/pbc.10461 14752789

[127] Kato I Kasukabe T Kumakura S. Menin−MLL Inhibitors Induce Ferroptosis and Enhance the Anti−Proliferative Activity of Auranofin in Several Types of Cancer Cells. Int J Oncol (2020) 57(4):1057–71. doi: 10.3892/ijo.2020.5116 32945449

[128] Chen RS Song YM Zhou ZY Tong T Li Y Fu M. Disruption of xCT Inhibits Cancer Cell Metastasis *via* the Caveolin-1/Beta-Catenin Pathway. Oncogene (2009) 28(4):599–609. doi: 10.1038/onc.2008.414 19015640

[129] Schmitt A Xu W Bucher P Grimm M Konantz M Horn H. Dimethyl Fumarate Induces Ferroptosis and Impairs NF-κb/STAT3 Signaling in DLBCL. Blood (2021) 138(10):871–84. doi: 10.1182/blood.2020009404 33876201

[130] Zhang Y Tan H Daniels JD Zandkarimi F Liu H Brown LM. Imidazole Ketone Erastin Induces Ferroptosis and Slows Tumor Growth in a Mouse Lymphoma Model. Cell Chem Biol (2019) 26(5):623–33.e9. doi: 10.1016/j.chembiol.2019.01.008 30799221 PMC6525071

[131] Kinowaki Y Kurata M Ishibashi S Ikeda M Tatsuzawa A Yamamoto M. Glutathione Peroxidase 4 Overexpression Inhibits ROS-Induced Cell Death in Diffuse Large B-Cell Lymphoma. Lab Invest (2018) 98(5):609–19. doi: 10.1038/s41374-017-0008-1 29463878

[132] Yang WS SriRamaratnam R Welsch ME Shimada K Skouta R Viswanathan VS. Regulation of Ferroptotic Cancer Cell Death by GPX4. Cell (2014) 156(1-2):317–31. doi: 10.1016/j.cell.2013.12.010 PMC4076414 24439385

[133] Xia X Fan X Zhao M Zhu P. The Relationship Between Ferroptosis and Tumors: A Novel Landscape for Therapeutic Approach. Curr Gene Ther (2019) 19(2):117–24. doi: 10.2174/1566523219666190628152137 PMC7046989 31264548

[134] Yu H Guo P Xie X Wang Y Chen G. Ferroptosis, a New Form of Cell Death, and Its Relationships With Tumourous Diseases. J Cell Mol Med (2017) 21(4):648–57. doi: 10.1111/jcmm.13008 PMC5345622 27860262

[135] Crombie J LaCasce A. The Treatment of Burkitt Lymphoma in Adults. Blood (2021) 137(6):743–50. doi: 10.1182/blood.2019004099 33171490

[136] Mancuso RI Foglio MA Olalla Saad ST. Artemisinin-Type Drugs for the Treatment of Hematological Malignancies. Cancer Chemother Pharmacol (2021) 87(1):1–22. doi: 10.1007/s00280-020-04170-5 33141328

[137] Zhao X Zhong H Wang R Liu D Waxman S Zhao L. Dihydroartemisinin and its Derivative Induce Apoptosis in Acute Myeloid Leukemia Through Noxa-Mediated Pathway Requiring Iron and Endoperoxide Moiety. Oncotarget (2015) 6(8):5582–96. doi: 10.18632/oncotarget.3336 PMC4467388 25714024

[138] Kim C Lee JH Kim SH Sethi G Ahn KS. Artesunate Suppresses Tumor Growth and Induces Apoptosis Through the Modulation of Multiple Oncogenic Cascades in a Chronic Myeloid Leukemia Xenograft Mouse Model. Oncotarget (2015) 6(6):4020–35. doi: 10.18632/oncotarget.3004 PMC4414170 25738364

[139] Wang N Zeng GZ Yin JL Bian ZX. Artesunate Activates the ATF4-CHOP-CHAC1 Pathway and Affects Ferroptosis in Burkitt’s Lymphoma. Biochem Biophys Res Commun (2019) 519(3):533–9. doi: 10.1016/j.bbrc.2019.09.023 31537387

[140] Kalkat M De Melo J Hickman KA Lourenco C Redel C Resetca D. MYC Deregulation in Primary Human Cancers. Genes (Basel) (2017) 8(6):151. doi: 10.3390/genes8060151 PMC5485515 28587062

[141] Vecchio E Fiume G Mignogna C Iaccino E Mimmi S Maisano D. IBTK Haploinsufficiency Affects the Tumor Microenvironment of Myc-Driven Lymphoma in E-Myc Mice. Int J Mol Sci (2020) 21(3):885. doi: 10.3390/ijms21030885 PMC7038122 32019112

[142] Carroll PA Freie BW Mathsyaraja H Eisenman RN. The MYC Transcription Factor Network: Balancing Metabolism, Proliferation and Oncogenesis. Front Med (2018) 12(4):412–25. doi: 10.1007/s11684-018-0650-z PMC7358075 30054853

[143] Adams JM Harris AW Pinkert CA Corcoran LM Alexander WS Cory S. The C-Myc Oncogene Driven by Immunoglobulin Enhancers Induces Lymphoid Malignancy in Transgenic Mice. Nature (1985) 318(6046):533–8. doi: 10.1038/318533a0 3906410

[144] Alexander WS Schrader JW Adams JM. Expression of the C-Myc Oncogene Under Control of an Immunoglobulin Enhancer in E Mu-Myc Transgenic Mice. Mol Cell Biol (1987) 7(4):1436–44. doi: 10.1128/mcb.7.4.1436-1444.1987 PMC365231 3037318

[145] Petrenko O Li J Cimica V Mena-Taboada P Shin HY D’Amico S. IL-6 Promotes MYC-Induced B Cell Lymphomagenesis Independent of STAT3. PloS One (2021) 16(3):e0247394. doi: 10.1371/journal.pone.0247394 33651821 PMC7924759

[146] Eischen CM Weber JD Roussel MF Sherr CJ Cleveland JL. Disruption of the ARF-Mdm2-P53 Tumor Suppressor Pathway in Myc-Induced Lymphomagenesis. Genes Dev (1999) 13(20):2658–69. doi: 10.1101/gad.13.20.2658 PMC317106 10541552

[147] Zindy F Eischen CM Randle DH Kamijo T Cleveland JL Sherr CJ. Myc Signaling *via* the ARF Tumor Suppressor Regulates P53-Dependent Apoptosis and Immortalization. Genes Dev (1998) 12(15):2424–33. doi: 10.1101/gad.12.15.2424 PMC317045 9694806

[148] Langdon WY Harris AW Cory S Adams JM. The C-Myc Oncogene Perturbs B Lymphocyte Development in E-Mu-Myc Transgenic Mice. Cell (1986) 47(1):11–8. doi: 10.1016/0092-8674(86)90361-2 3093082

[149] Wang S Xu L Feng J Liu Y Liu L Wang J. Prevalence and Incidence of Multiple Myeloma in Urban Area in China: A National Population-Based Analysis. Front Oncol (2019) 9:1513. doi: 10.3389/fonc.2019.01513 32039008 PMC6993203

[150] Liu W Liu J Song Y Wang X Zhou M Wang L. Mortality of Lymphoma and Myeloma in China, 2004-2017: An Observational Study. J Hematol Oncol (2019) 12(1):22. doi: 10.1186/s13045-019-0706-9 30832702 PMC6399942

[151] Siegel RL Miller KD Jemal A. Cancer Statistics, 2019. CA Cancer J Clin (2019) 69(1):7–34. doi: 10.3322/caac.21551 30620402

[152] Gozzelino R Arosio P. Iron Homeostasis in Health and Disease. Int J Mol Sci (2016) 17(1):130. doi: 10.3390/ijms17010130 PMC4730371 26805813

[153] Bordini J Galvan S Ponzoni M Bertilaccio MT Chesi M Bergsagel PL. Induction of Iron Excess Restricts Malignant Plasma Cells Expansion and Potentiates Bortezomib Effect in Models of Multiple Myeloma. Leukemia (2017) 31(4):967–70. doi: 10.1038/leu.2016.346 27881873

[154] Tu BP Weissman JS. Oxidative Protein Folding in Eukaryotes: Mechanisms and Consequences. J Cell Biol (2004) 164(3):341–6. doi: 10.1083/jcb.200311055 PMC2172237 14757749

[155] Shimizu Y Hendershot LM. Oxidative Folding: Cellular Strategies for Dealing With the Resultant Equimolar Production of Reactive Oxygen Species. Antioxid Redox Signal (2009) 11(9):2317–31. doi: 10.1089/ars.2009.2501 PMC2819804 19243234

[156] Bordini J Bertilaccio MT Ponzoni M Fermo I Chesi M Bergsagel PL. Erythroblast Apoptosis and Microenvironmental Iron Restriction Trigger Anemia in the VK*MYC Model of Multiple Myeloma. Haematologica (2015) 100(6):834–41. doi: 10.3324/haematol.2014.118000 PMC4450630 25715406

[157] Wu KJ Polack A Dalla-Favera R. Coordinated Regulation of Iron-Controlling Genes, H-Ferritin and IRP2, by C-MYC. Science (1999) 283(5402):676–9. doi: 10.1126/science.283.5402.676 9924025

[158] Gu Z Wang H Xia J Yang Y Jin Z Xu H. Decreased Ferroportin Promotes Myeloma Cell Growth and Osteoclast Differentiation. Cancer Res (2015) 75(11):2211–21. doi: 10.1158/0008-5472.Can-14-3804 PMC4946247 25855377

[159] NA A FH ME Naqishbandi AM Efferth T. Induction of Apoptosis, Autophagy and Ferroptosis by Thymus Vulgaris and Arctium Lappa Extract in Leukemia and Multiple Myeloma Cell Lines. Molecules (2020) 25(21):5016. doi: 10.3390/molecules25215016 PMC7663330 33138135

[160] Zhong Y Tian F Ma H Wang H Yang W Liu Z. FTY720 Induces Ferroptosis and Autophagy *via* PP2A/AMPK Pathway in Multiple Myeloma Cells. Life Sci (2020) 260:118077. doi: 10.1016/j.lfs.2020.118077 32810509

[161] Bordini J Morisi F Cerruti F Cascio P Camaschella C Ghia P. Iron Causes Lipid Oxidation and Inhibits Proteasome Function in Multiple Myeloma Cells: A Proof of Concept for Novel Combination Therapies. Cancers (Basel) (2020) 12(4):970. doi: 10.3390/cancers12040970 PMC7226326 32295216

